# A high-quality apple genome assembly reveals the association of a retrotransposon and red fruit colour

**DOI:** 10.1038/s41467-019-09518-x

**Published:** 2019-04-02

**Authors:** Liyi Zhang, Jiang Hu, Xiaolei Han, Jingjing Li, Yuan Gao, Christopher M. Richards, Caixia Zhang, Yi Tian, Guiming Liu, Hera Gul, Dajiang Wang, Yu Tian, Chuanxin Yang, Minghui Meng, Gaopeng Yuan, Guodong Kang, Yonglong Wu, Kun Wang, Hengtao Zhang, Depeng Wang, Peihua Cong

**Affiliations:** 1 Key Laboratory of Biology and Genetic Improvement of Horticultural Crops, Research Institute of Pomology, Chinese Academy of Agricultural Science, 125100 Xingcheng, Liaoning China; 2 Nextomics Biosciences Institute, 430000 Wuhan, Hubei China; 3 USDA-ARS National Center for Genetic Resources Preservation, Fort Collins, CO 80521 USA; 4 Beijing Agro-Biotechnology Research Center, Beijing Academy of Agriculture and Forestry Sciences, 100097 Beijing, China; 5 Zhengzhou Fruit Research Institute, Chinese Academy of Agricultural Science, 450009 Zhengzhou, Henan China

## Abstract

A complete and accurate genome sequence provides a fundamental tool for functional genomics and DNA-informed breeding. Here, we assemble a high-quality genome (contig N50 of 6.99 Mb) of the apple anther-derived homozygous line HFTH1, including 22 telomere sequences, using a combination of PacBio single-molecule real-time (SMRT) sequencing, chromosome conformation capture (Hi-C) sequencing, and optical mapping. In comparison to the Golden Delicious reference genome, we identify 18,047 deletions, 12,101 insertions and 14 large inversions. We reveal that these extensive genomic variations are largely attributable to activity of transposable elements. Interestingly, we find that a long terminal repeat (LTR) retrotransposon insertion upstream of *MdMYB1*, a core transcriptional activator of anthocyanin biosynthesis, is associated with red-skinned phenotype. This finding provides insights into the molecular mechanisms underlying red fruit coloration, and highlights the utility of this high-quality genome assembly in deciphering agriculturally important trait in apple.

## Abstract (web summary)

Existing apple genome assemblies all derive from Golden Delicious. Here, the authors combine different sequencing technologies to assemble a high quality genome of an anther-derived homozygous genotype HFTH1 and find the association of a retrotransposon and red fruit colour.

## Introduction

The apple genome is a foundation of genetic research and DNA-informed breeding that drives innovations for sustainable apple production^1,2^. Although the availability of the current high-quality genome of Golden Delicious and the resequencing of major genotypes enable rapid progress in apple genomics and breeding studies^3–6^, only a single reference genome together with short-read resequencing data presents some limitations in the discovery of new genes and characterisation of genomic variations, which may substantially contribute to genome evolution and the genetics of complex traits^3,7,8^. A large-scale survey of genomic variations will provide insights into the potential biological mechanisms of key traits, which in turn will aid the development of genetic markers for marker-assisted selection (MAS) breeding in apple. Recent studies have demonstrated that an extensive range of functional genomic variation can be readily uncovered through direct comparative analyses of the several high-quality genomes^9,10^. In addition, apple (*Malus domestica* Borkh.) is among the most diverse and economically important fruit species in the Rosaceae family^3,6^, but we still lack an in-depth understanding of genetic basis of its many economically important traits. In the case of red skin colouration, although the developmental and environmental regulatory mechanisms of the anthocyanin biosynthetic pathway have been well characterised and the corresponding genes have been identified^11–14^, the genetic basis for the regulation of fruits colouration is not yet fully understood. Dissection of the genetic determinants of this crucial trait appears to be difficult without the availability of high-quality genome sequences.

**Fig. 1:**
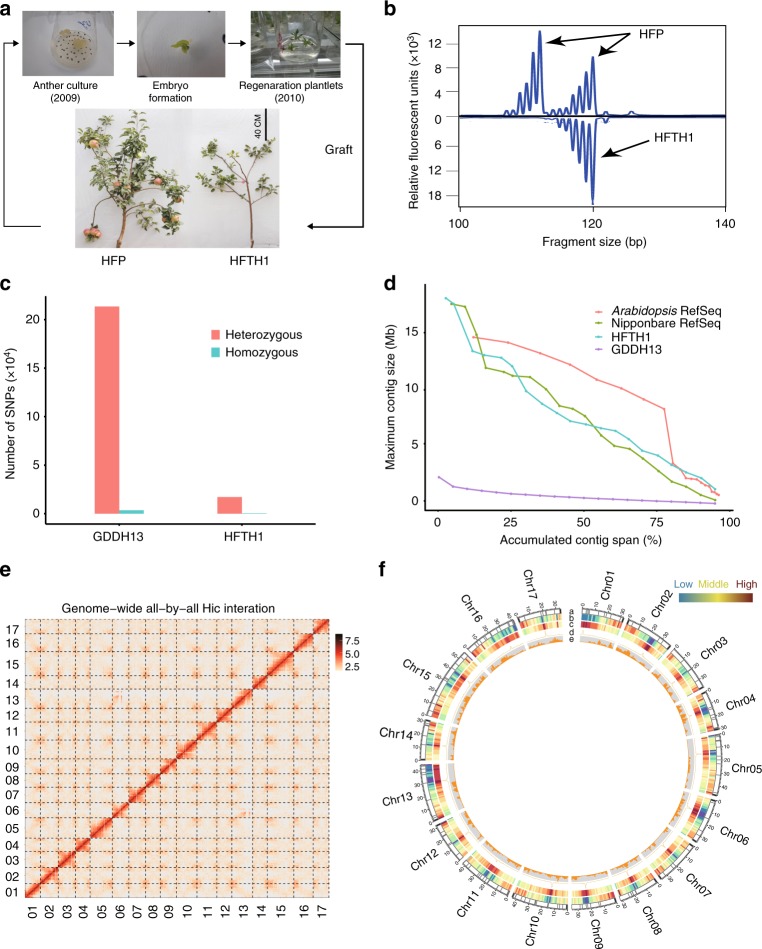
Overview of the assembly quality and characteristics of the HFTH1 genome. **a** Regenerated plantlets derived from Hanfu anther culture and the dramatic changes between the HFTH1 phenotype and the heterozygous donor (HFP) genotype. **b** Homozygosity analysis of chromosome 01 of HFTH1 and HFP using simple sequence repeats (SSRs, the results of the analysis of all chromosomes are shown in Supplementary Fig. 1). **c** Counts of SNPs detected in the GDDH13 and HFTH1 genomes. SNPs were detected using Illumina reads from the same individual that was used for the assembly. The heterozygous SNP (red) represents the heterozygosity of a genome and the homozygous SNP (blue) represents the potential error for an assembly. **d** Distribution of the contig length in the assemblies of *Arabidopsis* (TAIR10), Nipponbare (RGAP7), HFTH1 and GDDH13. Each contig size (*y*-axis) represents the minimum contig size that covered the cumulative percentage of the assembly (*x*-axis) after the assembled contigs were sorted from the largest to the smallest. **e** Hi–C interactions among 17 chromosomes with a 100-kb resolution. Strong interactions are indicated in dark red and weak interactions are indicated in yellow. **f** Circular diagram depicting the characteristics of the HFTH1 genome. The tracks from outer to inner circles indicate the following: **a** chromosomes (Chr.), gaps and telomeres, the black regions at the end of each chromosome represent assembled the telomere sequences and the grey regions represent gap regions; **b** gene density (window size of 1 Mb); **c** repeat density (window size of 1 Mb); **d** Copia-7; **e** Golden Delicious shared SNPs (window size of 500 kb)

All cultivated apple genotypes are a highly heterozygous and ancient autotetraploid of 17 chromosomes, presenting enormous challenges for genome analyses and breeding^3^. Thus, the anther-derived homozygous genotype HFTH1 was developed for sequencing (Fig. 1a). This homozygous line has the advantage of simplifying genome assembly^3^, and its parentage Hanfu (Dongguang x Fuji) is a very influential cultivar in China, that has several desirable traits, including bright red skin, cold-resistance and long shelf-life^15^. Here, we present a high-quality reference genome of HFTH1 using a combination of SMRT sequencing, Hi-C sequencing and optical mapping. We then use this genome to perform a comparative genomic analysis with the existing apple genomes. We track the highly dynamic evolution of transposons, and discover an LTR retrotransposon that is associated with the red-skinned phenotype and thus serves as a valuable tool for MAS breeding. This additional reference genome provides a foundation for functional genomics and transposon biology, and enhances our understanding of the genome variation that shapes phenotypic diversity in apple.

## Results

### Genome sequencing and assembly

To minimise the complexity of assembly, an anther-derived trihaploid Hanfu line (HFTH1) was used for genome sequencing, while its donor Hanfu was a highly heterozygous diploid cultivar (HFP, Fig. 1a, b and Supplementary Fig. 1). The homozygosity of the HFTH1 genome was confirmed by microsatellite markers and k-mer spectrum analysis (Fig. 1b and Supplementary Figs. 1, 2). The HFTH1 genome was determined as a triploidy by flow cytometry analysis, suggesting that HFTH1 had undergone spontaneous chromosome duplication during in vitro culture (Supplementary Fig. 3). Compared with the recently published genome of double haploid Golden Delicious line^3^ (GDDH13), HFTH1 was more homozygous, as demonstrated by calling heterozygous SNPs from Illumina reads obtained from the corresponding individual (Fig. 1c). This higher level of homozygosity is more favourable for improving the quality of the genome assembly.

Our sequencing of HFTH1 resulted in coverage of ~117-fold PacBio single-molecule long reads (77 Gb with an average length of 13.1 kb), 66-fold Illumina paired-end short reads (43.3 Gb), 224-fold optical map data (147.8 Gb with an average length of 178.9 kb) and 145-fold Hi-C data (95.5 Gb, Supplementary Table 1). The assembly was performed in a stepwise fashion^16^, and the initial assembly of the PacBio-only data generated a 656.52 Mb genome size with a contig N50 of 4.63 Mb (Supplementary Table 2). The initial contigs were polished with PacBio long reads and Illumina short reads. Subsequently, the polished contigs were scaffolded using optical map data, and during this step four contigs containing conflicting connections were identified and split to resolve conflicts, and 58.5% gaps that were introduced in this step were closed by subsequent gap filling procedure. Finally, scaffolding with Hi-C data allowed the accurate clustering and ordering of 17 pseudo-chromosomes covering the 658.90 Mb assembly, with a contig N50 of 6.99 Mb and a maximum contig length of 18.01 Mb (Supplementary Table 3 and Fig. 1d, e). The assembly size was close to the estimated genome size of GDDH13^3^, but represented 92.99% of our estimated genome size (708.54 Mb) for HFTH1 by k-mer analysis, and ~97.89% of the Illumina reads of HFTH1 could be mapped to our assembly (Supplementary Table 4). In addition, the 160,068 bp chloroplast genome and 396,939 bp mitochondria genome were assembled into two complete contigs (Supplementary Fig. 4).

### Assessment of genome quality

The quality and completeness of the assembly were evaluated using several different strategies. For the base accuracy of the sequencing, the quality value (QV) of the assembly was estimated to be at least 41, which compared very favourably with those of two published mammalian genomes^16,17^ (QV35 for gorilla and QV34.5 for goat) that were also assembled with PacBio data. For the structural accuracy of assembly, ~98.71% of the mapped Illumina reads of HFTH1 could be mapped with the correct orientation and estimated insert size, versus 94.36% of the mapped reads of GDDH13 (Supplementary Table 4). Furthermore, the whole-genome alignment of HFTH1 and GDDH13 showed strong collinearity and consistency (Supplementary Fig. 5).

Our assembly captured 22 long stretches of telomeric sequences (5′-TTTAGGG-3′) at both ends of seven chromosomes and at a single end of eight chromosomes, with repeat numbers ranging from 294 to 1073 (Table 1 and Fig. 1f). Moreover, based on a benchmark of 1440 conserved plant genes^18^, ~97.0% complete BUSCO genes and 98.02% of the expressed sequence tags (ESTs) of *Malus domestica* from GenBank could be detected in the assembly (Supplementary Tables 3 and 5), confirming the high completeness of the assembly. In addition, the contig N50 value of our assembly was comparable to that of the rice genome^19^ (RGAP7), and the HFTH1 genome covered approximately the same BUSCOs as the *Arabidopsis* genome^20^ (TAIR10) and RGAP7 genome (Supplementary Table 3 and Fig. 1d), which demonstrated that the level of completeness of our genome was at a similar to that of the model plant genomes, even though HFTH1 had the largest genome size and the highest proportion of repetitive sequences compared with the genomes of *Arabidopsis* and rice (Supplementary Table 3). The completeness of our genome provided an opportunity to comprehensively assess genome variations between HFTH1 and GDDH13.

**Table 1:** Statistics of HFTH1 and GDDH13 genome assemblies

Chromosome	HFTH1			GDDH13		
	Length (bp)	# Gaps	Telomere	Length (bp)	# Gaps	Telomere
Chr01	32,944,118	12	Single	32,625,452	85	Single
Chr02	38,449,405	8	Both	37,577,729	87	-
Chr03	37,138,690	4	Single	37,524,076	80	-
Chr04	31,012,745	7	Both	32,301,874	64	Single
Chr05	47,891,858	13	Single	47,952,461	107	-
Chr06	35,567,198	5	Both	37,137,259	88	Single
Chr07	35,934,761	5	Both	36,691,129	75	-
Chr08	31,511,015	7	Single	31,609,270	66	Single
Chr09	34,800,404	9	Single	37,604,908	79	Single
Chr10	43,815,736	13	Both	41,762,413	82	Single
Chr11	42,456,296	14	Single	43,059,885	90	-
Chr12	32,285,079	8	Single	33,050,054	74	-
Chr13	44,866,511	12	Single	44,339,518	118	Single
Chr14	31,515,206	5	Both	32,513,452	61	Single
Chr15	56,644,392	15	-	54,945,402	128	Single
Chr16	41,670,059	14	-	41,389,449	92	-
Chr17	33,998,825	7	Both	34,748,701	75	Single
mtDNA	396,939	0	NA	396,947	0	NA
cpDNA	160,068	0	NA	160,068	0	NA
Unanchored	7,992,922	326	-	52,728,359	839	-

*NA* not available

### Genome annotation

We identified 44,677 high-confidence protein-coding genes, and ~4.29% of annotated genes were located in the gap regions of the GDDH13 genome. The annotated genes covered 95.9% of the complete BUSCO genes (Supplementary Table 3), and ~92.28% of the annotated genes were expressed in at least one tissue or homologous to known proteins, which suggested that our genes annotation was very complete.

We employed a combination of de novo and homology-based approaches to annotate repetitive sequences. Approximately 393.88 Mb and 362.18 Mb transposable elements (TEs) were identified in the HFTH1 and GDDH13 genomes, respectively (Supplementary Tables 3 and 6). The difference in the repeat sequence content (~32 Mb) can account for 93.09% of the additional non-N bases between the HFTH1 and GDDH13 genomes. Although we were unable to identify any significantly enriched tandem centromeric repeat elements in the HFTH1 genome using existing approaches^21,22^, 17 blocks (one block per chromosome) with particularly high proportions of repeat elements ( >90%) in the HFTH1 genome, were located near the middle of chromosomes (Supplementary Table 7 and Fig. 1f). These regions can be assumed to represent part of putative heterochromatin regions on the HFTH1 chromosomes. The most abundant repeat element family in these regions was Copia-7 (ID from Repbase^23^), and most of the Copia-7 elements were concentrated in these regions (Fig. 1f). Although *HODOR* (a high-copy Golden Delicious repeat) was identified as the most repetitive consensus sequence in the apple genome^3^, the Copia-7 elements showed low similarity with *HODOR*.

### Gap filling for the reference Golden Delicious genome

Owing to the genomic congruence between GDDH13 and HFTH1, we used the HFTH1 genome to fill gaps in the GDDH13 genome, as a similar approach has been applied to the genomes of human^24^, gorilla^17^ and goat^16^. In total, 488 gaps (adjacent gaps were merged and counted as one gap) in the GDDH13 genome, with average and median lengths of 78,864 bp and 15,647 bp, were completely closed (Supplementary Fig. 6a). Among these, the longest gap closure was 2,859,572 bp and spanned 256 genes with 33.98% tandem duplicated genes. Approximately 97.75% of the closed gaps were located in repeat regions (Supplementary Fig. 6b), and most of these gaps had been assembled into multiple short fragments or filled with ambiguous (N) bases in previously published genomes^3–5^. In particular, ~39.34% sequences of the filled genomic gaps in GDDH13 could be found on its unanchored Chr00. For example, one 719,872 bp gap (the expected gap size was 728,347 bp) could be completely closed using the HFTH1 assembly, whereas this gap was assembled into 2 and 11 fragments in the assemblies reported by Velasco et al.^4^ and Li et al.^5^, respectively (Supplementary Fig. 6c). In addition, we filled nine genomic gaps with an average length of 42,360 bp for the HFTH1 genome using the GDDH13 genome. These additional sequences will help to improve genomic annotation and discover thousands of functional genes and regulatory elements.

### Genome comparison between Golden Delicious and Hanfu

Genome variations, such as insertions, deletions, inversions and duplications, are an important source of the genetic diversity that shapes phenotypic variations. Hanfu exhibits an obviously different phenotype from that of Golden Delicious, including red skin, a strong cold-resistant habit, a high resistance to alternaria leaf spot and branch ring-rot, long fruit storability, and an obvious short branching character^15^. The comparison of the Golden Delicious genome with our HFTH1 reference genome, allowed us to directly catalogue the extent of the genomic variation between these two cultivars.

First, an average density of 2.15 Golden Delicious shared SNPs per kilobase was identified (Fig. 1f). Approximately 3.34% of the SNPs were located within 31.69% of protein-coding genes containing non-synonymous substitutions. An enrichment analysis of the InterPro domains of genes with non-synonymous SNPs, showed that these genes were significantly correlated with the disease-resistant domains (Supplementary Table 8), which demonstrated that these genes may evolve under different selection pressures in two cultivars, and provide resistance to various environmental stresses.

**Fig. 2:**
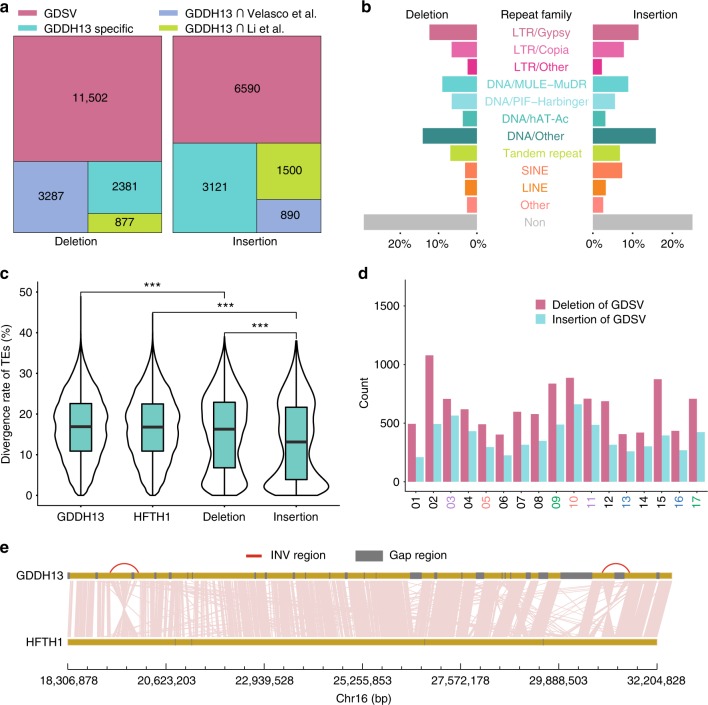
Characterisation of structural variants. **a** The overlap of structural variations ( > 100 bp) between GDDH13 and other published assemblies of Golden Delicious. **b** Classification of repeat elements associated with structural variations. A repeat element was defined as being associated with a structural variation if it overlaps with the structural variation. LTR, long terminal repeat; DNA, DNA transposon; SINE, short interspersed element; LINE, long interspersed element; Other, other types of repeat; Non, no repeats were detected. **c** Divergence rate of transposons of different sources. Deletion and insertion represent transposons associated with deletions and insertions, respectively. RepeatMasker was used to calculate the divergence rate from the consensus sequence of Repbase for each transposon. The middle hinge of all boxes is the median, the lower and upper hinges correspond to the 25th and 75th percentiles, and the whiskers represent the 1.5 inter-quartile range (IQR) extending from the hinges. Wilcoxon rank sum test, ***p < 0.001 and *n* = 435,202, 456,919, 6525, 4010 for GDDH13, HFTH1, Deletion, Insertion, respectively. **d** Count of structural variations detected in each chromosome. Duplicated chromosomes are shown in the *x*-axis with the same colour. **e** Synteny view of two large inversions with a length longer than 600 bp in chromosome 16 (19,782,372–20,450,041 and 31,388,523–32,032,862). Source data are provided in Source Data file 1

We identified 18,047 deletions and 12,101 insertions (including absence/presence variations) that were had a length greater than 100 bp in length. Of these, the presences of 63.73% of the deletions and 54.46% of the insertions in all published genomes of Golden Delicious (Fig. 2a), were defined as Golden Delicious shared structural variations (GDSVs). An enrichment analysis of the genes associated with GDSVs agreed with the results of the non-synonymous SNP analysis (Supplementary Table 9). For instance, one GDSV that includes a short interspersed nuclear element (SINE) was inserted in the 3′UTR of a disease-resistance gene (Supplementary Fig. 7a). Another GDSV was a deletion in the 5′UTR of a C-repeat/DRE binding factor (*MdCBF2*), which is a master regulator of cold acclimation^25^ (Supplementary Fig. 7b).

In addition, among the GDSVs, the average lengths of the deletions (508 bp) and insertions (519 bp) were very similar (Supplementary Fig. 8), and >70% of GDSVs were associated with TEs. The TE distribution patterns of insertions and deletions were similar, with the exception that the percentage of SINEs associated with insertion was approximately twofold higher than that found for deletions, which accounted for only 3–8% of GDSVs (Fig. 2b). We found that the divergence rates of TEs displayed a significant difference (*p*-value < 2.2e-16; Welch two-sample *t*-test), and that TEs associated with insertions were less divergent from the consensus TEs found in Repbase^23^ and younger than the TEs associated with deletions (Fig. 2c), which suggests that most of the Golden Delicious shared insertions were induced by young TEs insertion in the HFTH1 genome, after Hanfu and Golden Delicious diverged from a common ancestor.

Taking a whole-genome view of the distribution of SNPs and GDSVs, we found chr.15 had the highest average SNP density (2.99 per kilobase, Fig. 1f), whereas chr.2 exhibited the most structural variations (Fig. 2d). Most of the duplicated chromosomes from the same chromosome ancestor after a recent whole-genome duplication^4^ (WGD, chrs.3 and 11, 9 and 17, 13 and 16) showed similar contents of SNPs and GDSVs, whereas chrs.10 and 5 showed significantly different contents of SNPs (2.52:1, Fig. 1f) and GDSVs (1.66:1, Fig. 2d), particularly in one end of chr.5, which showed a lower diversity than the average diversity of whole-genome. However, chrs.10 and 5 had a similar gene content (Fig. 1f), suggesting that chr.5 may have undergone introgression and fixation during domestication and breeding process after the recent WGD.

Additionally, we identified 14 large inversions (INVs) with length longer than 100 kb, two of which on chr.16 were longer than 600 kb (Fig. 2e). We found that all the breakpoints of these two INVs were located within TE elements, which may have induced these inversion events.

### Dynamic evolution of LTR retrotransposons in Hanfu

**Fig. 3:**
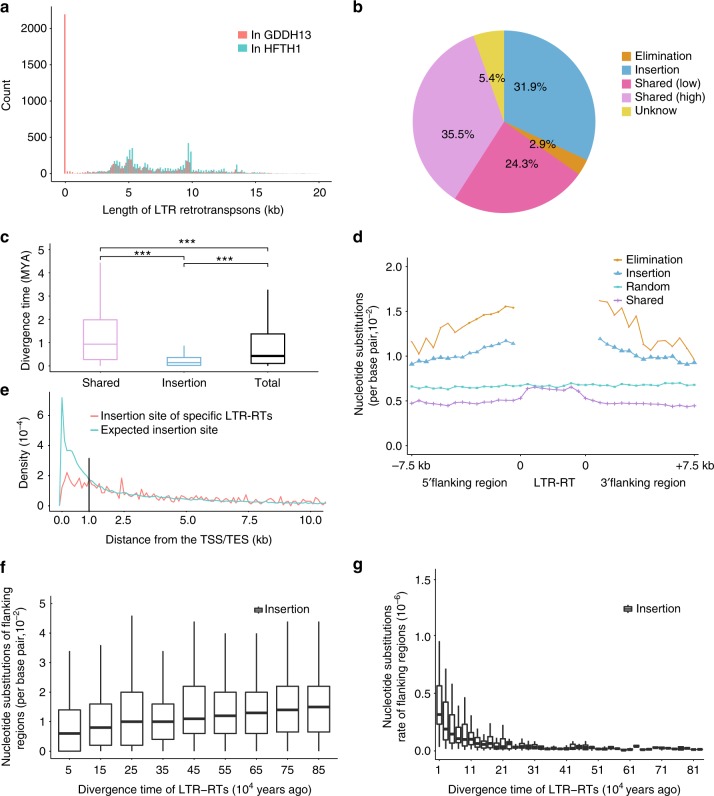
Evolution of intact LTR retrotransposons in the HFTH1 genome. **a** Length distribution of intact LTR-RTs from the HFTH1 genome. A large number of LTR-RTs in the GDDH13 genome have a length <100 bp, which indicated that these LTR-RTs were newly inserted into the HFTH1 genome or eliminated from the GDDH13 genome. **b** Pie chart showing the classification of the LTR-RTs in the HFTH1 genome (see Methods for the definition of each type). **c** Estimated divergence time of each category of LTR-RTs in the HFTH1 genome. The divergence time was calculated using a substitution rate of 1.3 × 10^−8^ substitutions per site per year. Wilcoxon rank sum test, ***p < 0.001 and *n* = 3468, 1850, 5800 for Shared, Insertion, Total, respectively. **d** Distribution of cumulative nucleotide substitutions of LTR-RTs and flanking sequences. The nucleotide substitutions of both flanking regions were calculated in a 400 bp sliding window, and the nucleotide substitutions of LTR-RT inside regions were calculated in ten non-overlapping equal bins. Three thousand blocks in the HFTH1 genome with a length 20 kb were randomly selected and used as a control. **e** Distribution of insert sites of specific LTR-RTs in the GDDH13 genome in 100-bp windows located at an increasing distance from genes. The distribution of intergenic distance was used as a control. **f** Distribution of cumulative nucleotide substitutions of flanking sequences over the divergence time of LTR-RTs. The flanking sequences were defined as 500 bp fragments located the upstream and downstream of LTR-RTs. The cumulative nucleotide substitutions were calculated in a sliding window of 100,000 years. **g** Rate of cumulative nucleotide substitutions of flanking sequences relative to the divergence time of LTR-RTs (see Methods for details). The middle hinge of all boxes is the median, the lower and upper hinges correspond to the 25th and 75th percentiles, and the whiskers represent the 1.5 inter-quartile range (IQR) extending from the hinges. Source data of Fig. 3a, b, c and e are provided in Source Data file 1. Source data of Fig. 3d, f and g are provided in Source Data file 2

TEs play an important role in driving adaptive evolution^7^, and >75% of the TEs from the apple genome were LTR-RTs. To track the highly dynamic evolution of LTR-RTs, we identified 7313 intact LTR-RTs with an average length of 7868 bp (Fig. 3a) in the HFTH1 genome. Approximately 62% of these LTR-RTs were present in the Golden Delicious genome based on its different assemblies (Fig. 3b and Supplementary Fig. 9), suggesting that these LTR-RTs may have been inserted in the common ancestor of the Hanfu and Golden Delicious lines. In the Rosaceae family, pear and apple share a highly conserved genomes. However, because of the fragmented genome assembly of the pear genome (contig N50 of 33.76 kb), only 40 intact LTR-RTs were found in the pear genome^26^ (Supplementary Fig. 9). Further analysis showed that most existing intact LTR-RTs in the apple genome may have been inserted after the divergence of apple and pear, which is in accordance with the findings that the average insert time (0.8 MYA) of these LTR-RTs was notably less than the estimated divergence time (8.1 MYA) of apple and pear (Fig. 3c and Supplementary Fig. 10). More than half of the shared LTR-RTs were highly similar between the HFTH1 and GDDH13 genomes (identity ≥0.99, Fig. 3b), and the average cumulative nucleotide substitutions (CNS) of the shared LTR-RTs and their flanking regions was less than the average level across the whole-genome (Fig. 3d) and most of the reverse-transcriptase domains (*gag, pol*, and *env*) of the shared LTR-RTs were not expressed in the ten tested samples (Supplementary Fig. 11a).

In addition, specific LTR-RTs (~31.9% of the total LTR-RTs) with two short target site duplications (TSDs) on their both sides, were only found on the HFTH1 genome (Fig. 3b), but only one of two TSDs was found in the corresponding position of the GDDH13 genome, suggesting that these specific LTR-RTs were inserted after the divergence of Hanfu and Golden Delicious. These specific insertion events, especially those near or inside genes, may be under strong selection for fixing, because protein-coding genes (coding plus intron regions) can account for 22.22% of whole-genome, but only 12.05% of the insertion events were located inside genes. The observed insertion events near genes were also lower than expected (Fig. 3e). Besides, the average expression level of genes near these insertion sites was less than that of the total gene set (Supplementary Fig. 11b). However, the selection pressure may be weakened or lost when the insertions located at least 1 kb from a gene (Fig. 3e). We found that 82.54% of specific LTR-RTs were expressed at least in one tested samples, compared with 64.39% of shared LTR-RTs (*p*-value < 2.2e-16; chi-squared test), which indicated that most specific LTR-RTs were young and active. The insertion events not only affect nearby gene expression, but also increase the mutation rate in the vicinity of the insertion site (~1.4- to 1.8-fold higher than the rate of the global nucleotide substitutions), which was in agreement with prior investigations^27,28^. Furthermore, the data also showed that the CNSs decreased as the distance from an LTR-RT increased; whereas the average CNSs in sequences surrounding shared inactive LTR-RTs was substantially lower (Fig. 3d). We found that the average CNSs increased gradually as the divergence time of LTR-RTs increased (Fig. 3f). Furthermore, the average CNSs rate appeared to slow and reach a bottleneck as the transposons were gradually inactivated and became other non-functional sequences (Fig. 3g). This result was consistent with the finding that most of the CNSs in the shared LTR-RTs were less than those of the specific LTR-RTs (Fig. 3d) even though the shared LTR-RTs had a greater evolutionary age (Fig. 3c). In addition, we found that ~2.9% of the LTR-RTs in the HFTH1 genome may have been eliminated in the GDDH13 genome, because 66.27% of these LTR-RTs had a length shorter than 100 bp at the corresponding site in the GDDH13 genome. In addition, the others had no blast hits (BLASTN *e*-values ≤ 10) to the corresponding LTR-RT sequence in the HFTH1 genome, and most of these sequences appear to be the remains of the transposition occurrence of other TEs in the GDDH13 genome (Fig. 3b). The average CNSs of the sequences surrounding these eliminated LTR-RTs was the highest in the genome (Fig. 3d), suggesting elimination events might have made a greater impact than insertions did during the evolution of TEs. The dynamic evolution of TEs likely created the unique genetic and phenotypic characteristics of HFTH1.

### Retrotransposon as an enhancer of *MdMYB1* expression

To evaluate the potential of our genome assembly for the genetic dissection of agriculturally important traits, we focused on red phenotype of apple fruit, which is a key determinant of consumer preference^12,13^. Genetic evidence has confirmed that *MdMYB1*, a core transcriptional regulator of the anthocyanin biosynthesis pathway, is responsible for the red phenotype^11,13,29^. In this study, we detected a significant difference between the transcript levels of *MdMYB1* and anthocyanin-related structural genes in the skins of ripening fruits of Hanfu and Golden Delicious by quantitative reverse transcription PCR (qRT-PCR) (Supplementary Fig. 12). *MdMYB1* had at least three types of alleles, namely, *MdMYB1-1*, *MdMYB1-2* and *MdMYB1-3*, and *MdMYB1-1* was a single dominant allele controlling anthocyanin synthesis in apple skin. The *MdMYB1-2* and *MdMYB1-3* alleles in non-red-skinned cultivars show a limited expression under intense light and low-temperature^12^. Furthermore, it has been shown that the coding region differences of these alleles do not affect their functional activity^13^. The reason for the significant differences in the expression levels among *MdMYB1* alleles has not been fully elucidated.

**Fig. 4:**
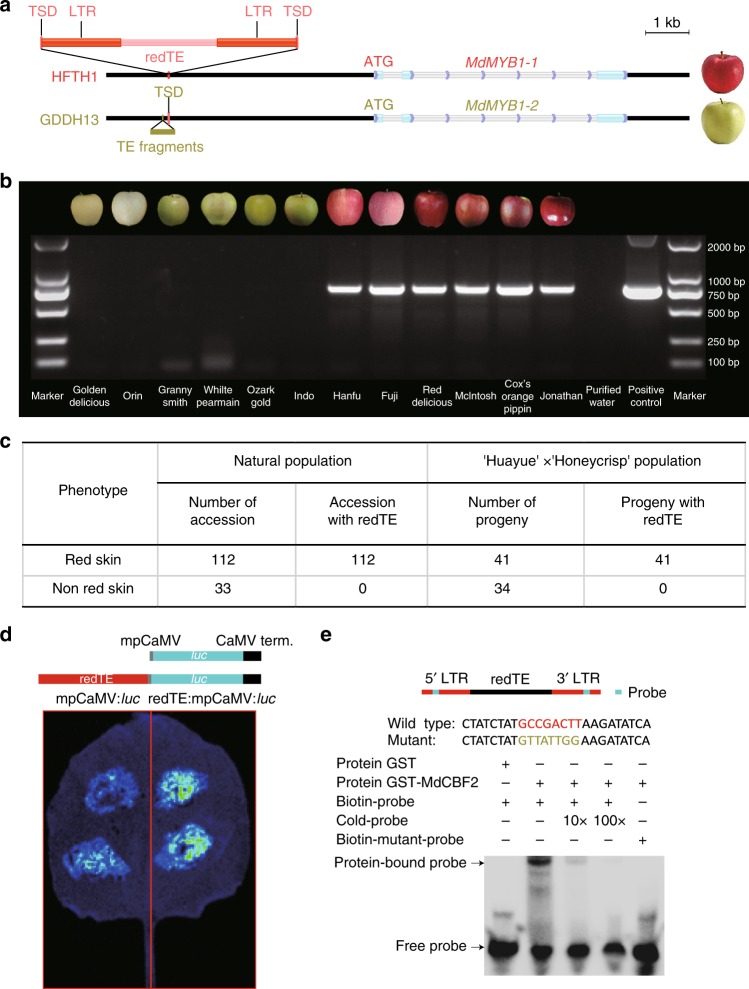
Red phenotype of apple associated with an LTR retrotransposon. **a** Molecular structure of *MdMYB1-1* and *MdMYB1-2* alleles with flanking sequences. The insertion sites upstream of *MdMYB1-1* and *MdMYB1-2* are indicated by a red line (HFTH1) and golden yellow line (GDDH13), respectively. **b** Images of 12 well-known apple varieties with non-red or red skin colour (upper panel) and PCR-based screen showing the absence (right) or presence (left) of the LTR retrotransposon insertion in the upstream of *MdMYB1*. A 750 bp fragment corresponding to the partial of redTE that is absent in non-red-skinned varieties (lanes 1 to 6) and is present only in red-skinned varieties (lanes 7 to 12). Lane 13, control check (purified water was used as the template), Lane 14, positive control. **c** PCR-based analysis of the redTE insertion in 145 accessions and the F1 segregating population from the cross of Huayue × Honeycrisp. **d** Constructs and transient expression assays showing that redTE obviously enhanced the luciferase expression levels. Upper panel, the mpCaM:*luc* (up) and redTE:mpCaM:*luc* (down) construct backbone consists of the minimal promoter from the cauliflower mosaic virus (mpCaMV, grey box), luciferase ORF and cauliflower mosaic virus terminator (black box). Lower panel, Luciferase image of *Nicotiana benthamiana* leaves 72 h after infiltration with the Agrobacterial strains containing mpCaM:*luc* (left), and redTE:mpCaM:*luc* (right), respectively. **e** MdCBF2 binds directly to the cis-acting element GCCGACTT. Source data of Fig. 4b, d are provided in Source Data file 1

The availability of two high-quality genomes allows a more precise comparative analysis. Specifically, we performed a sequence alignment of *MdMYB1* in the HFTH1 and GDDH13 genomes. The results showed that the coding sequences of *MdMYB1* were identical, but one SNP was detected in the intron regions. In addition, fifteen SNPs and five indels in the upstream region were found. Among of five indels, one 501 bp insertion occurs in the GDDH13 at a distance of −3394 bp upstream of the ATG initiation codon of *MdMYB1*, and is highly divergent from the neighbouring LTR-RTs (Fig. 4a); Another 4097 bp insertion is present in the HFTH1 at a distance of −3297 bp upstream of the ATG initiation codon of *MdMYB1*, and is a gypsy-like LTR retrotransposon (denoted redTE) with two TSDs (CATAT, Fig. 4a). Its two flanking LTR sequences (1274 bp) were completely identical, indicating that it was a recent insertion event. Although at least 3913 intact Gypsy-like retrotransposons were identified in the HFTH1 genome, only one of them had a 96.26% global identity with redTE (redTE-like, Supplementary Fig. 13), and the others showed <75.10% identity. Interestingly, redTE-like was only found in the HFTH1 genome, and not in GDDH13 through a genome-wide scan, and its two flanking LTR sequences (1262 bp and 1298 bp) harboured more mutations, suggesting that redTE-like was older than redTE.

To determine whether these differences were associated with fruits skin colour, we retrieved the sequences of these differential locis using data from public databases (Supplementary Table 10). The results showed that sixteen SNPs, two small indels in HFTH1 were also present in non-red-skinned accessions, and one 2-bp indel located in 17 repeat T bases in HFTH1 have a complex polymorphism in red apple accessions. In addition, the 501 bp insertion in the GDDH13 genome was also found in red-skinned accessions by PCR verification (Supplementary Fig. 14). Subsequently, we investigated the association of redTE with red skin colour, because it has been reported that retrotransposons often play crucial roles in tissue-specific expression patterns of pigment genes in plants^30^ and animals^31^. First, we retrieved resequencing data of the cultivars from GDR (ftp.bioinfo.wsu.edu, Supplementary Table 11) using the junction sequence GGATTTTTATATATGTGTTGACCCTA of redTE. The results showed that this target junction sequence were able to be found from the data of only red-skinned cultivars. Next, we screened 112 red-skinned accessions and 33 non-red-skinned accessions with known phenotypes using a PCR marker specific to redTE. The results showed that all the tested red-skinned accessions were completely associated with redTE, but redTE was not found in the non-red-skinned accessions (Fig. 4b, Supplementary Fig. 15 and Supplementary Data 1), suggesting that redTE insertion may be responsible for red phenotype in apple. Subsequently, we screened 75 progenies, including 41 with red skin and 34 without red skin, from the cross of Huayue (non-red skin) and Honeycrisp (red skin) (Fig. 4c, Supplementary Fig. 16 and Supplementary Data 2). The progenies analysis of this cross also confirmed the perfect co-segregation of redTE with red phenotype. These results suggested that redTE insertion was responsible for red phenotype in apple, which further supported the hypothesis of a single dominant mutation model underlying skin colour. Subsequently, because red-fleshed apple usually also have a red skin, we tested whether they also possess redTE insertion. To that end, three red-fleshed accessions with homozygous R6 genotypes were analysed by specific PCR marker, and the results showed that red-fleshed apples didnot harbour this retrotransposon insertion (Supplementary Fig. 15 and Supplementary Data 1), suggesting that the red skin of red-fleshed apples is caused by the constitutive expression of *MdMYB10* binding to R6 motifs of its own promoter in an auto regulatory-loop manner^8^, independent of redTE.

Some studies have shown that TEs are an abundant source of enhancer activity in plants^32^. Based on its inserted position, redTE appears to provide local enhancer activities that modulate the light-responsive gene expression of the *MdMYB1*. To definitively assess whether redTE acts as an enhancer, we performed transient assays in *Nicotiana benthamiana* leaves to test the effects of redTE on the gene expression of firefly luciferase. The results showed that the construct with redTE led to a significant increase in reporter gene expression, relative to the construct with the minimal promoter alone (Fig. 4d). This observed enhancement of gene expression by redTE is consistent with the known higher level of *MdMYB1* expression in red apple, which is similar to the role of a functional transposon (hopscotch) as a long-distance enhancer of *tb1* gene expression in maize^33^.

In addition, many red cultivars originating from bud sports with different red patterns, such as Hanfu and HanM, Gala and GaleGala, etc., are pretty commonly used in apple production^34^ (Supplementary Fig. 15 and Supplementary Data 1). These cultivars and their bud-sport mutations have redTE in our studies. It is well-known that transposon-induced epigenetic changes often affect the differential expression of neighbouring genes and create novel patterning^35,36^. Additionally, a recent research showed that striped pigmentation of Honeycrisp apple fruits is associated with hyper methylation in the promoter of *MdMYB1*^14^. Therefore, we selected the fruits of Hanfu (red stripe) and its sports HanM (fully red) at the same time (Supplementary Fig. 17a), and detected the DNA methylation status of redTE and the promoter region of *MdMYB1* using McrBC-PCR. The results showed that the Hanfu DNA was methylated in the MR3 and MR7 regions, in agreement with previous studies^14,34^. The redTE was heavily methylated in the MR8-MR11 regions, while the degree of methylation in Hanfu is much higher than that of HanM in the region MR12 (Supplementary Fig. 17b), which indicated that redTE-induced epigenetic changes may be associated with the variable colour patterns.

Given that redTE may control *MdMYB1* expression in red apple, we aimed to identify regulatory networks of redTE and other transcription factors under different environmental stresses. In blood orange, a retrotransposon controls the fruit-specific accumulation of anthocyanins in response to cold stress^30^. In apple, a relatively low ambient temperature can also promote fruits coloration, which is presumably regulated via the recruitment of cold acclimation-related transcription factors. In particularly, redTE contains the core cis-acting element (GCCGACTT) for cold acclimation transcription factor DREB/CBF binding^37^. We selected the low-temperature-inducible transcription factor MdCBF2 for electrophoretic mobility shift assay (EMSA)-based binding analysis. The result confirmed that MdCBF2 was capable of binding to the GCCGACTT element (Fig. 4e), which indicated it is potentially involved in fruit coloration through redTE regulatory networks under a relatively low ambient temperature.

## Discussion

A high-quality genome assembly is valuable for identifying structural variants, integrating phenotype–genotype associations, resulting in insights into the mode and tempo of genome evolution and elucidating the genetic architecture of important traits^9,10^. The generation of our reference genome provides a high-quality complement to the GDDH13 genome, demonstrating the utility of both whole-genome sequences for the accurate identification of the large and complex SVs, and providing a basis for the comparative genomic investigation of the unique biological characteristics and intraspecific genome diversity in apple. Our comparative genomic results indicate that dynamic changes in TEs can lead to large amounts of SVs, which might impact the genotypes. The discovery of redTE, a locus-specific LTR-RT insertion in the HFTH1 genome, will inspire researchers to further discuss the importance of TEs as a creator of major phenotypic variations. In fact, genome-scale analyses of TE-induced effects are only just beginning to be explored in humans and other crop species^32^. Therefore, the annotation of TEs in the HFTH1 and GDDH13 genomes will allow detailed studies of the functional effects and dynamic activity of more TEs polymorphisms in the long-term evolutionary background among different apple genotypes.

The evolution of fruits colour in apple is important and intriguing. In this study, our finding of a redTE insertion upstream of the *MdMYB1* promoter was associated with red colouration. Thus, redTE was regarded as an enhancer, controlling the development of red colouration by lowering the threshold value of the light response. Cultivars, without this enhancer, fail to effectively produce anthocyanin. This phenomenon could well explain why non-red-skinned cultivars still show a faint red colouration under intense light. In addition, red apple fruits display rich skin colours ranging from stripe to blush to dark red, and these diverse phenotypes are putatively the result of that redTE-mediated control the distribution patterns of anthocyanin through the creation genetic and epigenetic alleles to manipulate the function of *MdMYB1* under natural conditions. However, in apple, the mechanism through which redTE mediates how genetic and epigenetic variation contributes to red phenotypic diversity and adaptation to light changes remains to be investigated. Furthermore, site-specific TEs can also be used to trace the genetic relationships and origins^38^. A study of worldwide genetic diversity and pedigree records in apple estimated that one yellow-skinned cultivar Golden Delicious (1916) and four red-skinned cultivars [Coxs Orange Pippin (1850), Red Delicious (1880), Jonathan (1826) and McIntosh (1870)] were the core founders of modern apple breeding^39,40^. These four red-skinned cultivars contain redTE insertion (Fig. 4b), which suggests that they also had a common parental origin. Notably, the accession *M*. *sieversii* also contains redTE, suggesting the redTE likely originated from its supposed primary wild ancestor in Xinjiang (Supplementary Data 1). Although, a recent genome resequencing revealed *M. sieversii* in Xinjiang, China, is an ancient isolated ecotype not directly contributing to apple domestication^6^, it’s possible that early human activities spreaded *M*. *sieversii* with eye-catching red skin, from Xinjiang, China, to other geographical areas. The Chinese soft apple Huacaiping that was domesticated from Xinjiang wild apple with >2000-year cultivation history in China^41^, harbours redTE, which indicates that red apple once spreaded westward and eastward along the old Silk Road. This result appears to agree well with the results of breeding and geographical investigations of the origins and history of domesticated apple^41,42^. Subsequently, red apples from the redTE-induced mutation ancestor have become increasingly popular by artificial selection. Interestingly, a recent study of blood oranges^30^ showed that two different TE insertions produced similar effects in controlling the cold-induced expression of Ruby, which modulates fruit colour. This finding raises the possibility that other TE insertions are present in apple whose colouring effects have not been characterised. Here, it remains to be seen whether an analogous redTE insertion enhances MYB transcription in pear and other fruits in the Rosaceae.

Additionally, owing to the long and laborious selection process of apple breeding, the ultimate goal of assembled genome is to serve as a guideline in developing tools for MAS breeding in apple. We identified a surprisingly abundant number of structural variations that were dispersed across the whole-genome, which will be highly useful in developing functional markers for apple breeding. For instance, the redTE-based specific marker, is a particularly valuable tool for pre-selection of hybrid seedlings with objective skin colour in apple breeding programmers, because it is more efficient and precise than the previously available markers^11,13,43^. The marker may greatly reduce the costs of apple breeding by eliminating a large number of non-target hybrid seedlings. In addition, this genome of HFTH1 from a resistant parent may provide an excellent basis for development of markers of cold-resistance and disease-resistance, such as branch ring-rot and alternaria leaf spot in apple breeding^44^. Overall, this near-complete genome and other genomic resources will aid the mining of genes and functional markers and support the translation of research findings into genetic improvements for sustainable apple production.

## Methods

### Plant materials and DNA sequencing

The homozygous line HFTH1 was derived from an in vitro anther culture of a widely grown cultivar (HFP) in the cold region of northern China. HFTH1 and its donor parent, grafted on GM256 rootstock in 2010, were grown in the greenhouse and field at the Research Institute of Pomology, Chinese Academy of Agricultural Science. The DNA of HFTH1 was extracted from young leaves using the phenol-chloroform method. Two libraries with insert sizes of 300 bp and 20 kb were separately constructed using Illumina TruSeq Nano DNA Library Prep Kits and SMRTbell Template Prep Kits, and the 300 bp and 20 kb libraries were subsequently sequenced using an Illumina HiSeq X Ten instrument and a PacBio RS II instrument with the P6-C4 sequencing reagent, respectively. To obtain the BioNano optical mapping data, DNA was extracted from fresh young leaves of HFTH1, and embedded in a thin agarose layer for labelling at Nt.BspQI sites using the IrysPrep Reagent Kit protocol and subjected to optical scanning on the BioNano Irys platform. The Hi-C library, including cellular crosslinking, chromatin digestion, labelling of DNA ends, DNA ligation, purification and fragmentation, was constructed using the standard procedure as follows. Firstly, nuclear DNA from young leaves was cross-linked in situ, extracted, and digested with a restriction enzyme. The sticky ends of the digested fragments were biotinylated, diluted, and then ligated randomly. The biotinylated DNA fragments were enriched and sheared again to generate sequencing library, which was subsequently sequenced on Illumina HiSeq 4000 (http://en.annoroad.com/).

### Genome assembly

Falcon^45^ (v0.4) was used for constructing initial contigs using the following parameters: length_cutoff = 13,000 length_cutoff_pr = 14,000 pa_DBsplit_option = -x1000 -s250 -a pa_HPCdaligner_option = -v -dal128 -t12 -e.75 -k20 -h320 -l1800 -s1000 falcon_sense_option = --output_multi --min_idt 0.75 --min_cov 2 --local_match_count_threshold 2 --max_n_read 400 --output_dformat ovlp_DBsplit_option = -x1000 -s200 ovlp_HPCdaligner_option = -v -dal100 -t12 -k18 -h280 -e.96 -l1800 -s1000 overlap_filtering_setting = --max_diff 50 --max_cov 80 --min_cov 2 --bestn 10. The initial polishing was performed with Quiver^46^ using PacBio-only long reads, and then Pilon^47^ (v1.20) was utilised to further correct the PacBio-corrected contigs with accurate Illumina short reads. The BioNano data was first assembled to a consensus map using the IrysView software with a molecular length threshold of 150 kb and a minimum labels per molecule of 8, and hybrid scaffolding of the PacBio-corrected contigs and BioNano-based consensus map was performed using the hybrid scaffolding module within IrysView software with manufacturer’s suggested parameters. After scaffolding, PBJelly from PBSuite^48^ (v14.9.9) was performed to close gaps in the hybrid assembly. We re-performed error correction procedures to polish the sequences in the gap regions. Subsequently, the mitochondrial and chloroplast scaffolds or contigs were removed through alignment to mitochondrial and chloroplast references of apple, and any scaffolds or contigs for which at least 80% of the total length was aligned and that showed an identity larger than 90% were discarded as mitochondrial or chloroplast sequences. The Hi-C sequencing data were first aligned to the assembled genome using the bowtie2 end-to-end algorithm^49^, then the assembled scaffolds are clustered, ordered and directed onto the pseudo-chromosomes using Lachesis^50^. Finally, the pseudo-chromosomes predicted by Lachesis were cut into bins with equal length of 100 kb and used to construct a heatmap based on the interaction signals that generated by valid mapped read pairs to do validate and correct manually.

To obtain the genome of two organelles, we first used BLASR to map all raw PacBio long reads to the organelle references of *Malus* (downloaded from GenBank, accession NC_018554, NC_031163, KU851961, KX499859 and KX499861), any reads with a length longer than 20 kb, for which at least 80% of the total length were aligned and that showed an identity greater than 70% were used for the next assembly. The chloroplast genome was assembled with Canu^51^ (v1.3) (genome size = 160k) and the mitochondrial genome was assembled with Falcon (v0.4), the following error correction was then performed with the above-described procedures using Quiver and Pilon.

### Annotation of repeats

The repetitive sequences, including tandem repeats and TEs, in the HFTH1 and GDDH13 genomes were searched. First, we used Tandem Repeats Finder^52^ (TRF, v4.09) to annotate the tandem repeats using the following parameters: 2 7 7 80 10 50 2000. Then TEs were identified at both the DNA and protein levels using a combination of de novo and homology-based approaches. At the DNA level, LTR_FINDER^53^ (v1.0.6) was first used to identify LTR-RTs and RepeatModeler^54^ (v1.0.5) was utilised to construct a de novo repeat library, which comprised a repeat consensus database with classification information. We employed RepeatMasker^54^ (v4.0.6) to search for similar TEs in the known Repbase TE library^23^, MIPS Repeat Element Database^55^ (v9.3) and de novo repeat library. At the protein level, RepeatProteinMask within the RepeatMasker package was used to search against the TE protein database using a WU-BLASTX engine.

Telomere sequences were identified by searching both ends of the pseudo-chromosomes for high copy number repeats with the repeat unit 5-TTTAGGG-3. Putative heterochromatin regions were identified by searching for 1 Mb windows (250 kb step) with >90% repeat elements and the adjacent centromere windows were merged.

### Gene prediction and annotation

The MAKER pipeline^56^ (v2.31.8), which incorporates ab initio prediction, homology-based prediction and RNA-Seq assisted prediction, was used to annotate gene models. The protein sequences used for homology-based prediction were from five sequenced plants, namely, *Arabidopsis thaliana* (https://www.arabidopsis.org/index.jsp)*, Prunus persica* (https://www.rosaceae.org/)*, Pyrus communis* (https://www.rosaceae.org/)*, Malus domestica* (GDDH13, https://iris.angers.inra.fr/gddh13/the-apple-genome-downloads.html), and *Fragaria vesca* (https://www.rosaceae.org/) and a total of 1440 benchmarking universal single-copy orthologues of embryophyta within the BUSCO software (v3.0.1), were initially mapped onto the HFTH1 genome using tBlastn. Subsequently, Exonerate^57^ (v2.2.0) were used to polish the BLAST hits and thereby acquire exact intron/exon positions. The transcriptional data, including three tissues of the HFTH1 and seven tissues from HFP, were assembled with Histat2^58^ (v2.05) and StringTie^59^ (v1.3.0), and the results were used to identify candidate exon regions, donor and acceptor sites. The repeat regions in the HFTH1 genome were first soft-masked, and MAKER was then run twice. First, we ran MAKER with only transcriptional data to generate imperfect gene models, which were used to train the parameters for SNAP^60^ (V2006-07-28) and Augustus^61^ (v3.2.2). All data and predictions were then used to produce a consensus gene set. We removed a gene model from the consensus gene set if it had a MAKER-defined annotation edit distance (AED) score higher than 0.5 and lacked transcript data or homologous protein support. To obtain a more complete gene set, we added the genes predicted by Augustus that had transcriptional data or homologous proteins support but were not included in the initial gene set, to the final gene set.

Gene functions were assigned according to the best match by aligning the protein sequences to the Swiss-Prot and TrEMBL databases^62^ using Blastp (with a threshold of *E*-value ≤ 1*e*^−5^). The motifs and domains were annotated using InterProScan^63^ (v5.24) by searching against publicly available databases, including ProDom, PRINTS, Pfam, SMRT, PANTHER and PROSITE. The Gene Ontology (GO) IDs for each gene were assigned according to the corresponding InterPro entry.

### RNA sequencing and data analysis

RNA was extracted from seven tissues of HFP and three tissues of HFTH1 using the Quick RNA Isolation Kit (Cat.No.046-50QK, Huayueyang Biotechnology Beijing Co.Ltd., http://www.huayueyang.com/) and then characterised by agarose gel electrophoresis and a Nano Drop ND1000 spectrophotometer (Nano Drop Technologies, Wilmington, DE, USA). The complementary DNA (cDNA) libraries were constructed as follows, the first step involves purifying the poly-A containing mRNA molecules using oligo-dT attached magnetic beads. Then, the mRNA is fragmented into small pieces using divalent cations. The cDNA were synthesised using the cleaved RNA fragments as templates. The cDNA fragments then go through an end repair process, the addition of a single A base, and then ligation of the adapters. The products are then purified and enriched with PCR to create the final cDNA library, and are sequenced on an Illumina HiSeq 4000. RNA reads were first mapped to the HFTH1 genome using HISAT2, and gene expression was then measured in fragments per kilobase of exon per million fragments mapped (FPKM) using StringTie. The counts of the mapped reads of LTR-RTs were calculated with BEDTOOLS^64^ (v2.23.0).

### Phylogenetic analysis

The sequences of protein-coding genes from HFTH1 and seven plants (*A. thaliana, F. vesca, R. occidentalis* (https://www.rosaceae.org/)*, P. mume* (downloaded from GeneBank, GCA_000346735.1)*, P. persica, P. communis* and *M. domestica*) were used for a gene family clustering analysis. First, Blastp was used to generate pairwise protein sequence with an *E*-value cutoff of 1*e*^−5^. Second, OrthoMCL^65^ (v2.0.9) was used to cluster genes with an inflation value of 1.5. The protein sequences from 1499 single-copy gene families found in more than eight species were extracted and aligned using MAFFT^66^ (v7.058), and the alignment was then back-translated to the nucleotide alphabet using PAL2NAL^67^ (v14). The poorly aligned positions and divergent regions of the alignment were eliminated using Gblocks (v0.91b, molevol.cmima.csic.es/castresana/Gblocks.html). Phylogenetic analysis was performed using a maximum likelihood (ML) method implemented in RaxML^68^ (v8.0.19) with the GTRGAMMA substitution model and 100 nonparametric Bootstrap replicates. *A. thaliana* was selected as the out-group. The divergence time for eight species was estimated based on fourfold degenerate sites from the filtered alignment. The Markov chain Monte Carlo algorithm for Bayes estimation was adopted to estimate the divergence time using MCMCTree within the PAML package^69^ (v4.6). The calibration time for the divergence between *Rosaceae* and *A. thaliana* (97~109 Mya) was obtained from the TimeTree database (http://www.timetree.org/).

SynMap (CoGe, http://www.genomevolution.org) was used to detect the conserved synteny blocks using homologous gene pairs with the following parameters: Maximum distance between two matches (-*D*): 20; Minimum number of aligned pairs (-*A*): 10; Algorithm (Quota Align Merge) with maximum distance between two blocks (-Dm): 500. Circos^70^ was used for visualisation.

### Gap filling

A modified protocol based on the method reported by Shi et al.^24^ was used. Briefly, using BEDTOOLS, gap regions were extracted from the GDDH13 genome, and adjacent gaps (distance ≤ 500 bp) were merged as one gap. The 500 bp fragments located upstream and downstream of each gap region were extracted and aligned to the HFTH1 genome using BWA^71^ with parameter -*a*. A gap was considered to be closed, if (i) both fragments aligned successfully (coverage ≥ 80%) within 500 kb on the same scaffold or chromosome with the same orientation; (ii) the total alignment positions of both fragments were <5 (both fragments in repeat regions were avoided); (iii) the intervening sequence between aligned positions of both fragments did not contain any ambiguous (N) bases (only consider closed gaps); (iv) the intervening sequences can be aligned successfully (coverage ≥ 80%) with any Golden Delicious genomes or >90% of the intervening sequence with effectively covered (depth >3) using 30-fold Illumina reads from GDDH13, the intervening sequences were polished using Pilon with the Illumina reads of GDDH13. The ambiguous (N) bases of gap regions in the GDDH13 genome were replaced with the polished intervening sequences. The gaps in the HFTH1 genome were filled with the GDDH13 genome using the same above-described method.

### Detection and analysis of genome variation

SNP detection was performed using BWA and Genome Analysis Toolkit^72^ (GATK, v3.8) with the following filtering options: Quality by depth (QD) < 2.0, Fisher strand (FS) > 60.0, RMS mapping quality (MQ) < 40.0, MQRankSum < −12.5, ReadPosRankSum < −8.0, Maximum depth (DP) > 360. An SNP was defined as Golden Delicious shared SNP if the SNP was detected in the Golden Delicious genomes of GDDH13, Velasco et al. and Li et al. but not detected in the HFP genome. Owing to the lack of Illumina reads in the genome reported by Velasco et al., we extracted 500 bp fragments located upstream and downstream of each SNP and aligned these to the genome reported by Velasco et al. to assess whether this SNP was shared. A functional analysis of SNPs was performed using the ANNOVAR software^73^.

Structural variants (SVs) were identified using BWA and Sniffles^74^ (v1.0.7). First, we mapped the PacBio-corrected long reads (corrected with FALCON during the assembly step) of HFTH1 to the GDDH13 genome using BWA-MEM (using the -*M* and -*x* parameters), and Sniffles was then used to identify indels with length > 100 bp and large inversions with length > 100 kb. To reduce false-positive SV results as much as possible, we extracted sequences from 500 bp upstream to 500 bp downstream of each indel from the GDDH13 genome and aligned them to the HFTH1 genome using BWA-MEM with the parameter -*a*; If the fragment was aligned successfully and included a large insertion or clip (corresponding to a deletion of SV), and a large deletion or gap (corresponding to an insertion of SV), this SV was retained in the final result, and the border and length of this SV were recalculated based on the alignment. Long inversions were confirmed by whole-genome alignment using MUMmer^75^ (v3.07).

Presence/absence variations (PAVs) were detected by whole-genome alignment using MUMmer with the parameter -maxmatch (using the GDDH13 genome as the reference). Using BEDTOOLS, we merge adjacent aligned blocks (distance ≤ 50 bp) and extracted unaligned regions with lengths longer than 100 bp. For some unaligned regions with ambiguous (N) bases, we removed or split these regions according to the positions of the N bases. These unaligned regions were further filtered using their average depth. First, BWA was used to map the Illumina short reads from GDDH13 and HFTH1 to the HFTH1 and GDDH13 genomes, respectively. The depth was calculated using SAMTOOLS^76^ (v1.2). If an unaligned region had an average depth <10% of the average depth of the whole-genome, this region was regarded as a PV. The PVs in GDDH13 were treated as deletions, and the PVs in HFTH1 were treated as insertions to ensure consistency with indels for further analysis.

### Detection and analysis of LTR-RTs

The LTR_retriever pipeline^77^ was conducted to identify intact LTR-RTs of the HFTH1 genome from the outputs of LTRharvest^78^ (parameters: -similar 90 -vic 10 -seed 20 -seqids yes -minlenltr 100 -maxlenltr 7000 -mintsd 4 -maxtsd 6 -motif TGCA -motifmis 1) and LTR_FINDER (Parameters:-*D* 15,000 -*d* 1000 -*L* 7000 -*l* 100 -*p* 20 -*M* 0.9) with default parameters. Owing to the high copy number of LTR-RTs, we extracted 500 bp fragments in the upstream and downstream of each LTR-RT. We first aligned both fragments back to the HFTH1 genome using BWA-MEM with parameter -*a*. If both fragments were uniquely aligned within 20 kb (the length of the longest LTR-RT) on the same scaffold and chromosome with the same orientation, this LTR-RT was retained (31 LTR-RTs were removed). We then aligned both fragments to the GDDH13 genome with the same parameters and filter criterion (1482 LTR-RTs were removed), and 5800 LTR-RTs were used for further analysis. The intervening sequences between the aligned positions of both fragments in the GDDH13 genome were aligned to the corresponding LTR-RT sequence in the HFTH1 genome using Blastn with parameter -*F* *F*. Different types of LTR-RT were distinguished using the following criteria: if (i) the length of the intervening sequence was > 100 bp and existed blast hits to the corresponding LTR-RT sequence in the HFTH1 genome, this LTR-RT was defined as Shared. The Shared type was further classified as high similarity (similarity ≥ 99%) and low similarity (similarity < 99%) with the global similarity; (ii) if two copies of the TSDs flanked the LTR-RT in the HFTH1 genome while only one TSD (no intervening sequences) existed at the corresponding site of the GDDH13 genome, this LTR-RT was defined as Insertion; In contrast, (iii) if the intervening sequence had no blast hits to the corresponding LTR-RT sequence in the HFTH1 genome and two or no TSDs were found at the corresponding site of the GDDH13 genome, this LTR-RT was defined as Elimination; (iv) other remaining LTR-RTs were defined as Unknown.

The cumulative nucleotide substitution rate (*V*) of flanking sequences of each LTR-RT was calculated as *V* = (*S*_f_ – *S*_c_)/*T*_ltr_ + *S*_c_/(2 × *T*_div_), where *S*_f_ is the cumulative nucleotide substitution frequency of flanking sequence, and it is equal to the number of cumulative nucleotide substitutions in the flanking sequence divided by the length of flanking sequence (500 bp); *S*_c_ is the cumulative nucleotide substitution frequency of the control region (500 bp) that was defined as a non-functional region (2 kb) with 9 kb away from the LTR-RT; *T*_ltr_ is the divergence time of LTR-RT; *T*_div_ is the divergence time between Golden Delicious and Hanfu.

### Amplification of the partial redTE and 501 bp insertion

The two primers (5′-GGTCACCCAACCCACACTGGGCCTTG-3′ and 5′-CGGCCGCAATCGCAAGACGCAGA-3′) were used for amplifying partial sequence of redTE, and the two primers (5′-GGATACATGCACTATTGATGCGCT-3′ and 5′-GGGAGTGTGATATCCGACAGTGTGTCT-3′) were used for amplifying 501 bp deletion sequence. Amplification was performed in a thermal cycler (Bio-Rad, C1000, USA), and the temperature programme consisted of an initial denaturation of 3 min at 95 °C followed by programmed for 32 cycles of denaturation at 98 °C for 10 s, annealing at 62 °C for 20 s and extension at 72 °C for 30 s, and final extension was for 2 min at 72 °C. The products were analysed by electrophoresis in 1.5% agarose gel containing ethidium bromide and photographed under a UV transilluminator (Azure C150, USA). The PCR product of 750 bp for redTE and 562 bp for 501 bp insertion were confirmed by Sanger sequencing.

### Luciferase reporter assays in *Nicotiana benthamiana* leaves

The two primers (5′-GGTACCTTATATATGTGTTGACCCTAGAAACT−3′ and 5′- GGAAGCTTACGAGCCGAAGCTCAA−3′) were used for amplifying redTE, and it was cloned upstream of the 35 S minimal promoter at the *Kpn*I–*Hind*III sites in pGreenII 0800-LUC vector, generate the reporter construct redTE:minimal 35S:*LUC*. A reporter construct containing the cauliflower mosaic virus (CaMV) 35S minimal promoter driving expression of the firefly luciferase gene was used to test the control region segments. Two reporter constructs were transformed into *Agrobacterium tumefaciens* strain GV3101. Bacterial suspensions were infiltrated into young leaves of the 8-week-old *N. benthamiana* plants using a needleless syringe. After infiltration, plants were grown first under dark for 12 h and then with 16 h light/8 h dark cycle for 60 h at 25 °C. The leaves were sprayed with 100 mM luciferin and maintained under dark condition for 2 min. The LUC images were captured in a low-light cooled CCD imaging apparatus (Tanon 5200Multi, China). The experiments were repeated independently at least three times with similar results.

### McrBC-based methylation assay

A McrBC-PCR method was used to analyse the methylation degree of in the MdMYB1 promoter and redTE regions. Briefly, one microgram of genomic DNA (gDNA) isolated from Hanfu and HanM peel samples were digested overnight with McrBC (New England Biolabs), with three biological replicates. The digested gDNA and its respective control were used as the template for semi-quantitative PCR analysis. The *MdMYB1* promoter and the redTE sequences were divided into seven and five fragments, respectively, amplified with their corresponding primers (Supplementary Table 12, MR1-MR7 are quoted from reference^34^) and visualised under the UV Transilluminator (Azure C150, USA) after 1.5% gel electrophoresis.

### Electrophoretic mobility shift assays

The coding region of MdCBF2 (MD06G1072200) was cloned into the pGEX4T-1 vector, and its recombinant vector was transformed into Rosetta (DE3) for induction expression. The electrophoretic mobility shift assays (EMSA) were performed using the LightShift Chemiluminescent EMSA Kit (#89880; Thermo Scientific), according to the manufacturer’s protocol. The unlabelled probes, biotin-labelled probes and biotin-labelled mutant probes at the 3′ end were synthesised by Genewiz Co., Ltd (Supplementary Table 13). The protein-DNA samples were separated on 6.5% acrylamide gels, and transferred to a nylon membrane, and signals were captured using ChemiDoc MP Imaging System (BIO-RAD).

### Reporting Summary

Further information on experimental design is available in the Nature Research Reporting Summary linked to this article.

## Supplementary information

Supplementary Information

Peer Review File

Reporting Summary

Description of Additional Supplementary Files

Supplementary Data 1

Supplementary Data 2

Source Data 1

Source Data 2

## Data Availability

The repeat annotation and gap filling scripts are available through [https://github.com/moold/Genome-data-of-Hanfu-apple].

## Data availability

Data generated during the study are deposited in the NCBI under study PRJNA482033. Raw data (PacBio and Illumina reads) have been deposited in the Sequence Read Archive (SRA) under study accession number SRX4557792, SRX4557793 and SRX4557794. RNA-seq data of ten tested samples from Hanfu are available under the SRA accession numbers SRX4557795-SRX4557802, SRX4557790, and SRX4557791 . Genome assembly and annotation data has been deposited at DDBJ/ENA/GenBank under the accession RDQH00000000. The version described in this paper is version RDQH01000000. FASTA files of chromosomes and genes, as well as gff files for gene models can also be downloaded from [https://github.com/moold/Genome-data-of-Hanfu-apple]. Data supporting the findings of this work are available within the paper and its Supplementary Information files. A reporting summary for this Article is available as a Supplementary Information file. The datasets generated and analysed during the current study are available from the corresponding author on reasonable request. The source data underlying Figs. 2, 3a, 3b, 3c, 3e, 4b and 4d, as well as Supplementary Figs. 3, 6, 11b, 12, 14, 15, 16 and 17 are provided in Source Data file 1. The source data underlying Figs. 3d, 3f, and 3g are provided in Source Data file 2.
