# Supplementary material for: A high-quality apple genome assembly reveals the association of a retrotransposon and red fruit colour
Source: Nat Commun. 2019 Apr 2;10:1494. doi: 10.1038/s41467-019-09518-x (PMC6445120; doi:10.1038/s41467-019-09518-x)
Supplement: Supplementary file 1 — Supplementary Information [file 41467_2019_9518_MOESM1_ESM.pdf]

A high-quality apple genome assembly reveals the association of a retrotransposon and red fruit colour

Zhang *et al.*

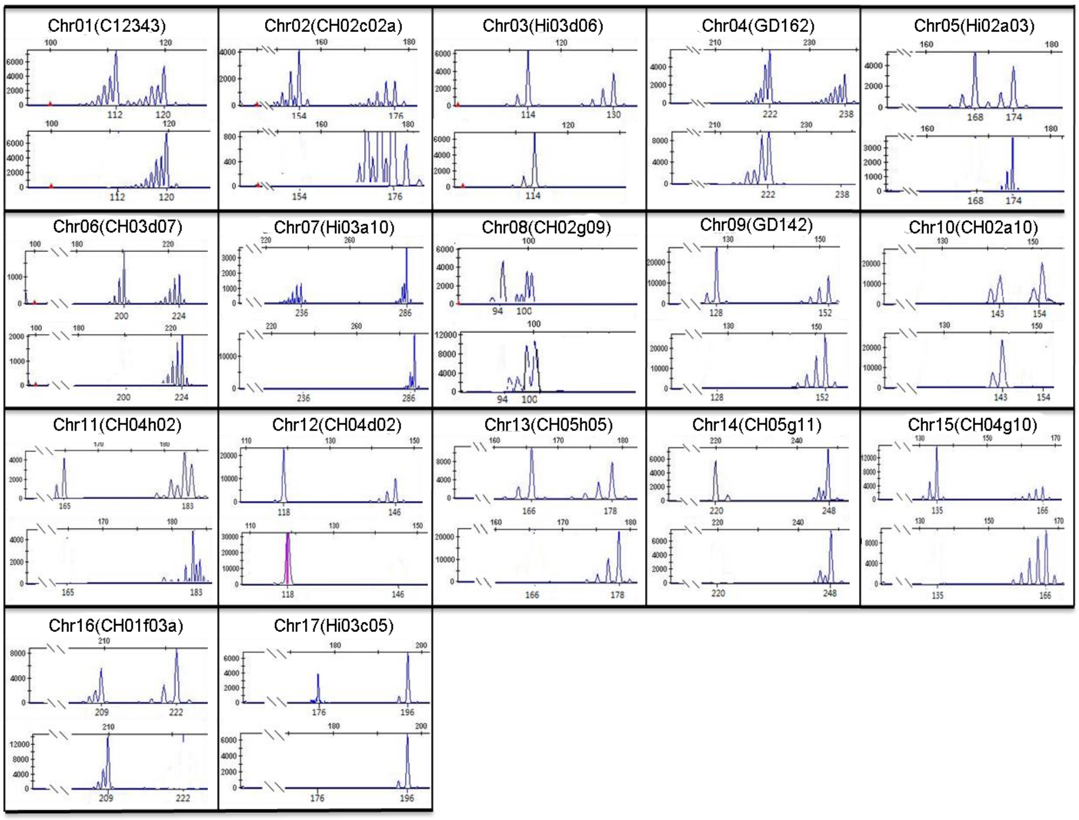

Supplementary Figure 1. Homozygosity analysis of HFTH1 and HFP using simple sequence repeats (SSRs) markers from the HIDRAS database (http://www.hidras.unimi.it/) by capillary electrophoresis with fluorescence detection. The x-axis shows the fragment size (bp) and the y-axis shows the peak height.

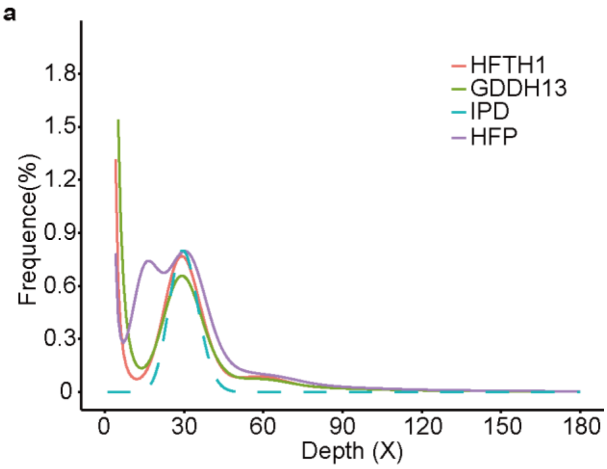

Supplementary Figure 2. Homozygosity analysis of the HFTH1 and GDDH13 genomes using Illumina reads. In the 17-mer depth distribution, the x-axis represents the k-mer depth, and the y-axis represents the frequency of k-mers. IPD, ideal Poisson distribution. HFP, heterozygous donor of HFTH1.

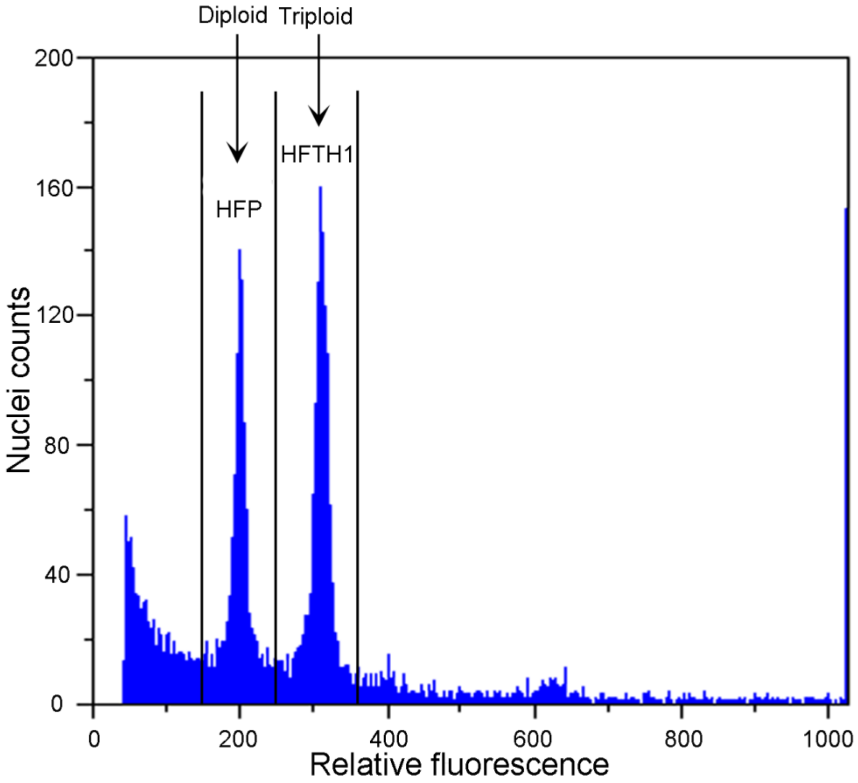

Supplementary Figure 3. Ploidy identification of HFTH1 and HFP by flow cytometry analysis. Source data are provided in Source Data file 1.

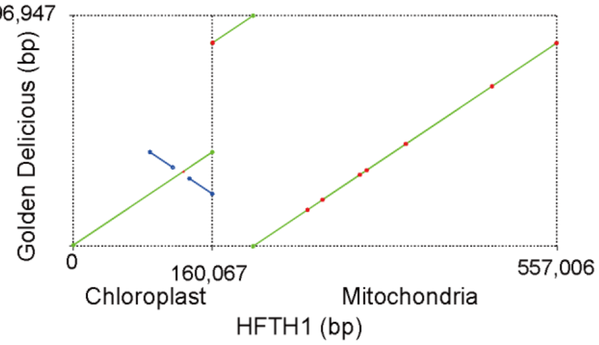

Supplementary Figure 4. Synteny view of two organelle genomes from Hanfu and Golden Delicious. The blue lines (left panel) represent two identical copies of a large inverted repeat (IR) sequence in the chloroplast genome. The red points (right panel) represent SNPs or indels at the current position. One point may contains multiple adjacent SNPs or indels.

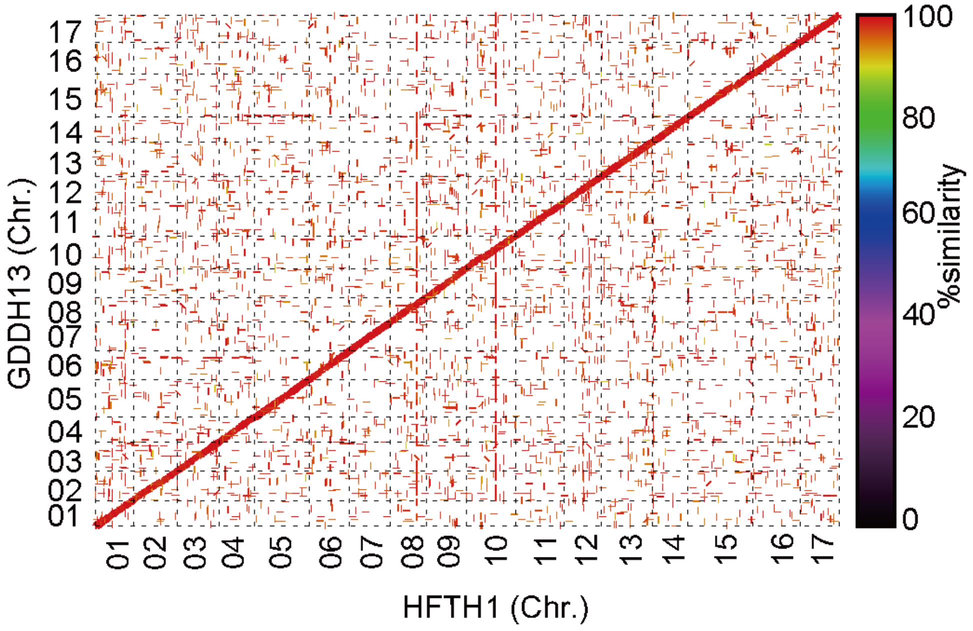

Supplementary Figure 5. Synteny view of the HFTH1 and GDDH13 genomes reveals a high degree of assembly consistency.

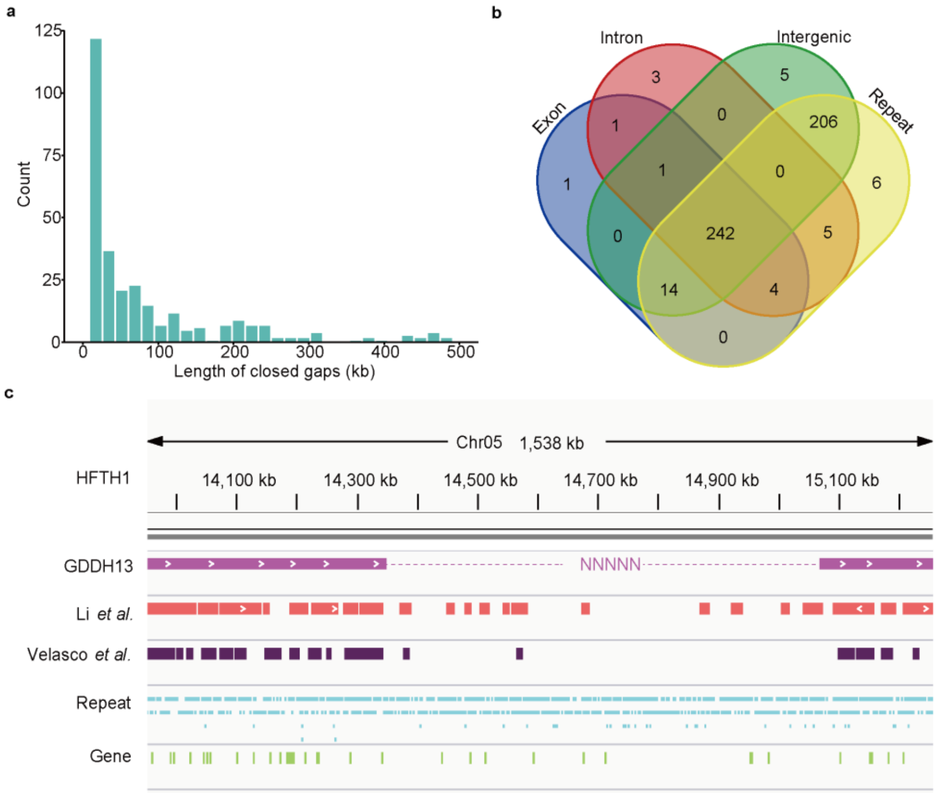

Supplementary Figure 6. Summary of gap filling in the GDDH13 genome. **(a)** Length distribution of all closed gaps in the GDDH13 genome. **(b)** Venn diagram of different classes of closed gaps in the GDDH13 genome. The class of a closed gap was defined basing on its overlapping elements. **(c)** Integrative Genomics Viewer screenshot of a 719,872 bp closed gap in the GDDH13 genome. Source data are provided in Source Data file 1.

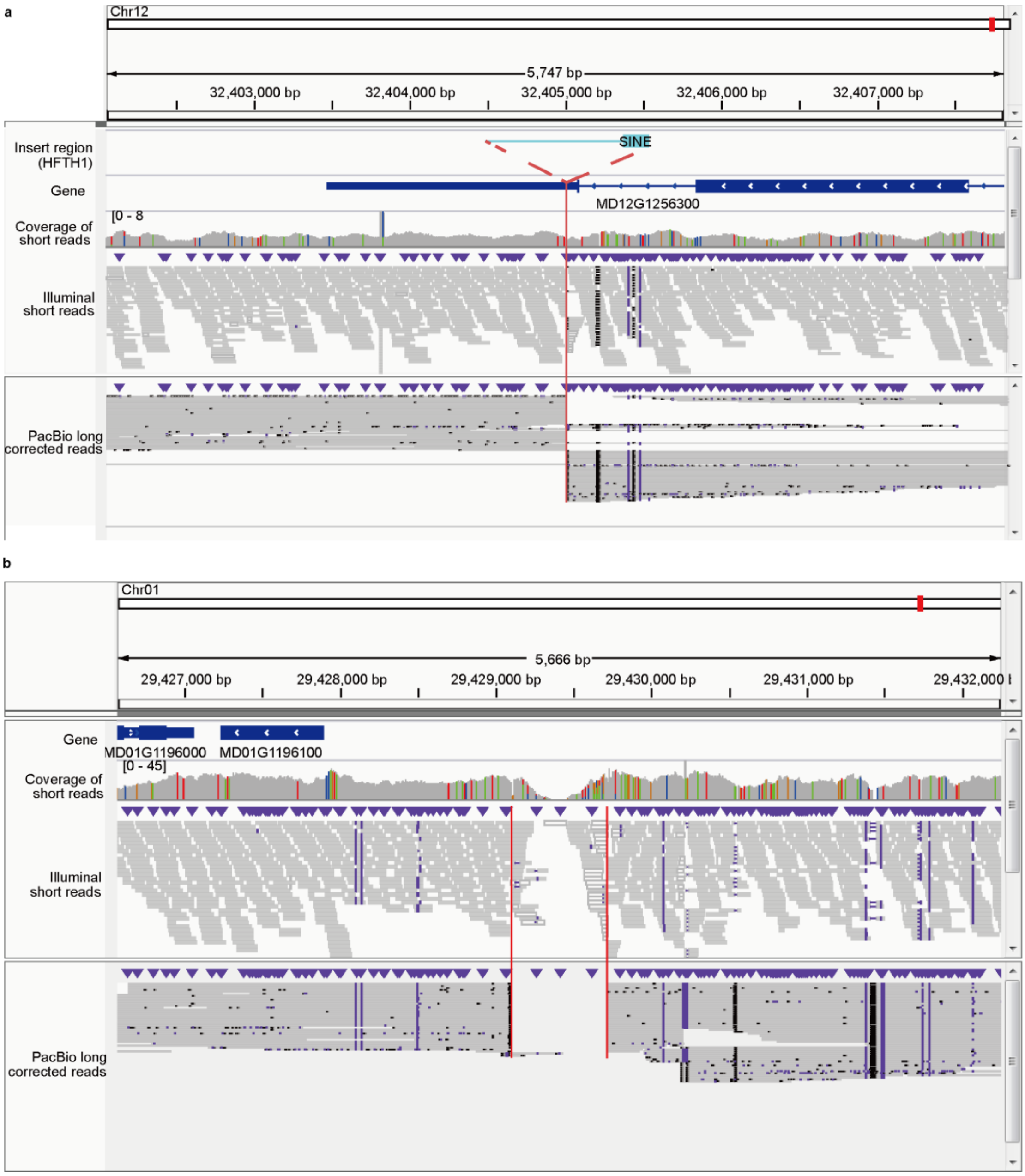

Supplementary Figure 7. Integrative genomics viewer screenshot of two structural variants. **(a)** Integrative genomics viewer screenshot of a 1,049 bp insertion in the GDDH13 genome. The PacBio and Illumina reads were from the HFTH1 genome, and the inserted sequence of the HFTH1 genome is shown at the top panel. **(b)** Integrative genomics viewer screenshot of a 625 bp insertion in the GDDH13 genome. PacBio and Illumina reads were obtained from the HFTH1 genome.

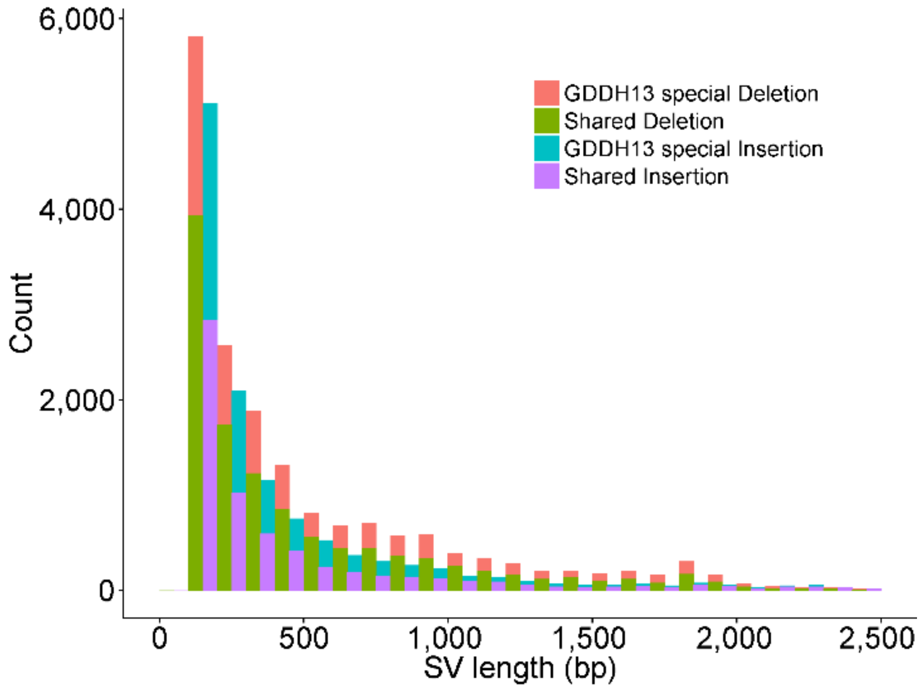

Supplementary Figure 8. Length distribution of structural variations. A special structural variation (deletion/insertion) indicates that it was only detected in the GDDH13 genome, while a shared structural variation (deletion/insertion) indicates that it was detected in all published genomes of Golden Delicious.

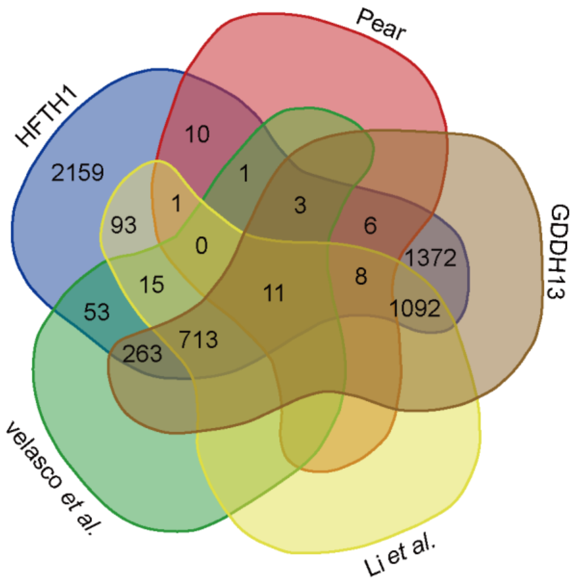

Supplementary Figure 9. Venn diagram showing the LTR-RTs in the HFTH1 genome amongst previously published assemblies of Golden Delicious and pear. The shared LTR-RTs may be underestimated because of the fragmented assembly.

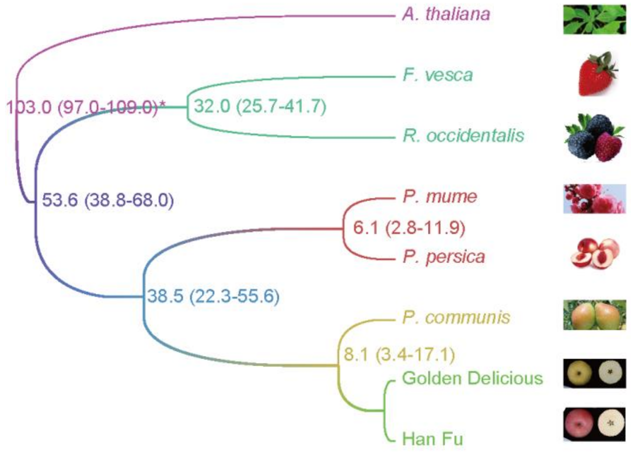

Supplementary Figure 10. Estimation of divergence time. The scale is in millions of years and 95% highest posterior density intervals are shown in brackets. The fossil-based divergence of *A. thaliana* and Rosaceae (103 MYR) was used for the calibration.

a

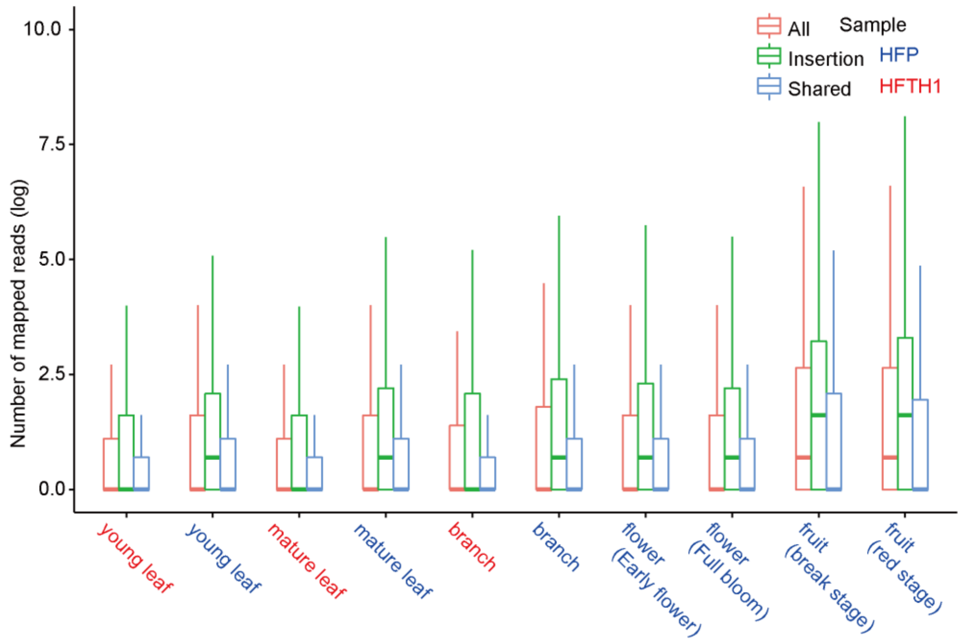

b

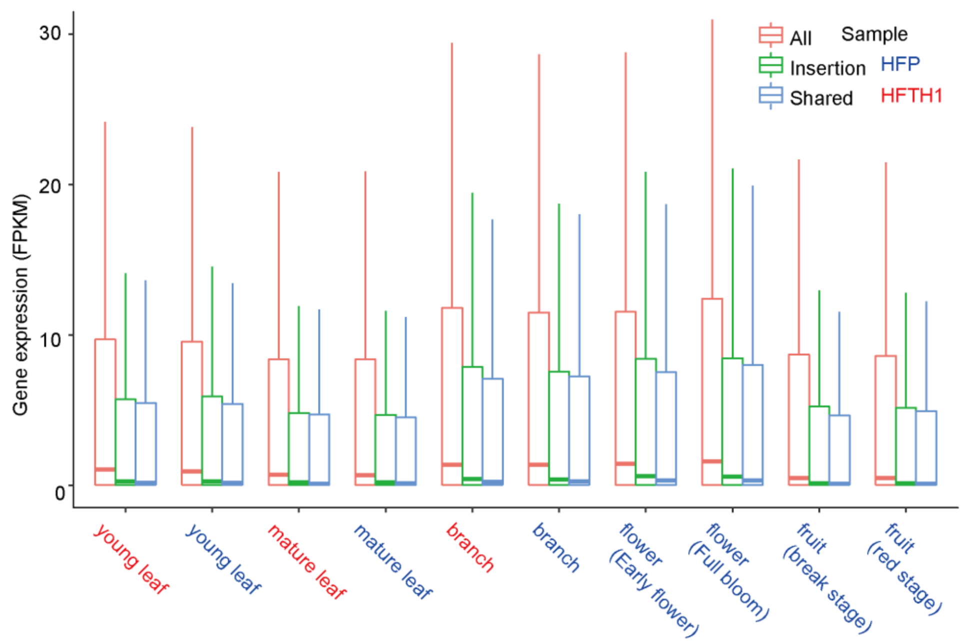

Supplementary Figure 11. Expression of LTR-RTs and nearby genes. **(a)** Expression of different types of LTR-RTs in 10 samples, and all represents the total LTR-RTs. **(b)** Expression of genes near LTR-RTs in 10 samples. Only the genes located within 5kb from LTR-RTs were selected. All represents the total gene set. The lower and upper hinges of all boxes correspond to the 25th and 75th percentiles; the whiskers represent the 1.5 inter-quartile range (IQR) extending from the hinges. Source data of Supplementary Data 11b are provided in Source Data file 1.

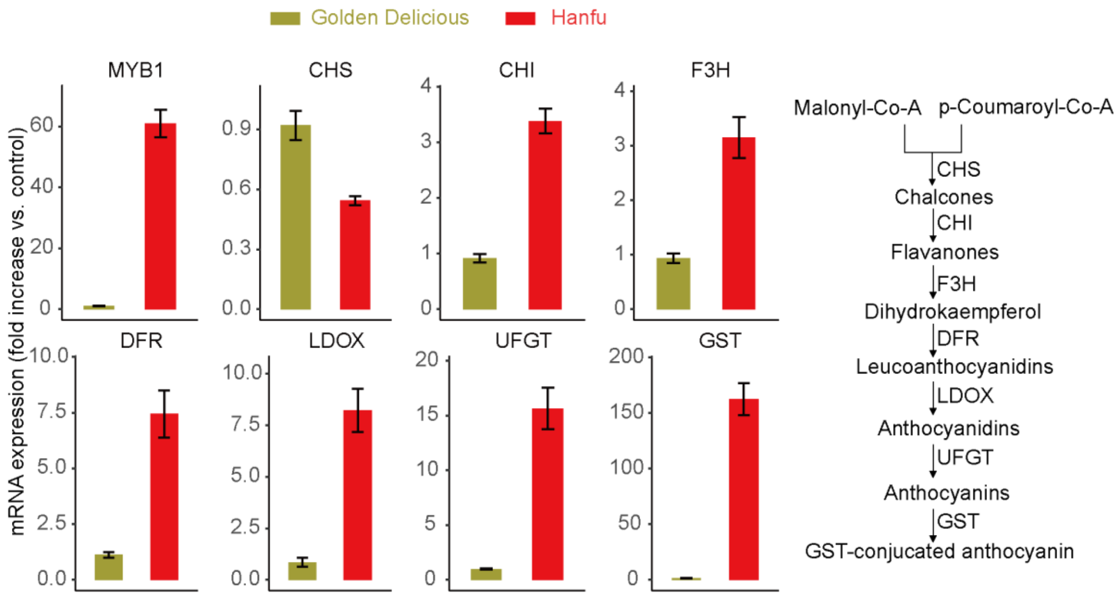

Supplementary Figure 12. Expression levels (left panel) of *MdMYB1* and structural genes involved in the anthocyanin biosynthetic pathway (right panel) in the peel of ripening fruits of Golden Delicious and Hanfu. CHS, chalcone synthase; CHI, chalcone isomerase; F3H, flavanone-3 b-hydroxylase. DFR, dihydroflavonol-4-reductase; LDOX, leucoanthocyanidin dioxygenase; UFGT, UDP-glycose flavonoid-3-O-glycosyltransferase; and GST, glutathione S-transferase. The mean and s.d. values from three independent experiments are shown. *MdActin* was used as a control. Source data are provided in Source Data file 1.

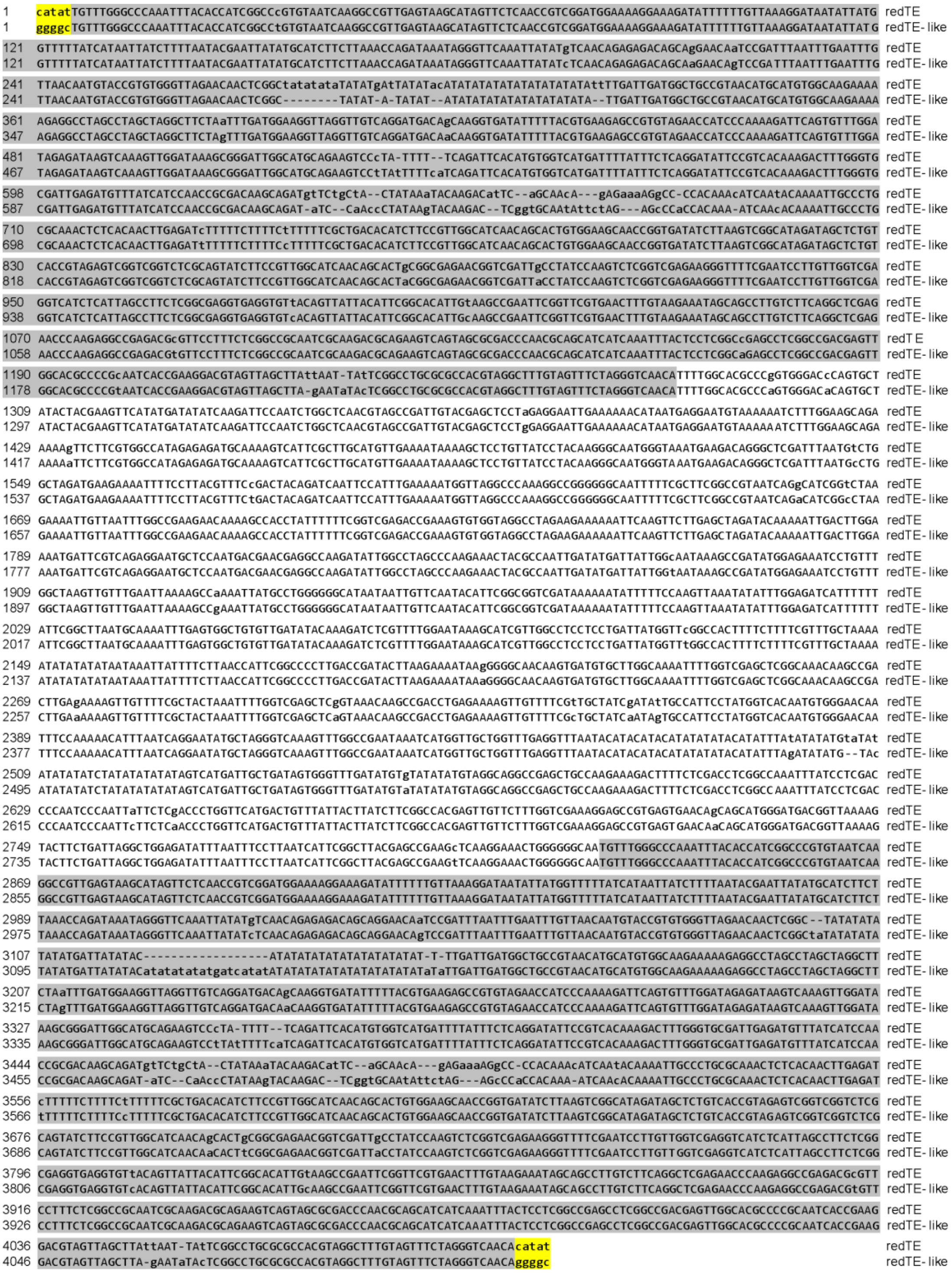

Supplementary Figure 13. Sequence alignment of redTE and redTE-like from HFTH1. 5-bp target site duplications are coloured in yellow, the grey indicates the two flanking long terminal repeats of redTE and redTE-like. Matched bases are shown in uppercase, and others are shown in lowercase.

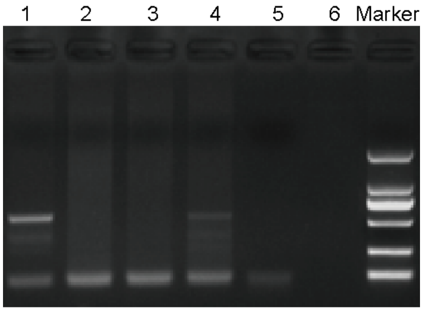

Supplementary Figure 14. PCR analysis of the 501 bp insertion. 1, McIntosh; 2, Hanfu; 3, Huayue; 4, Golden Delicious; 5, Water as template; 6, Blank; Maker, DL2000. Source data are provided in Source Data file 1.

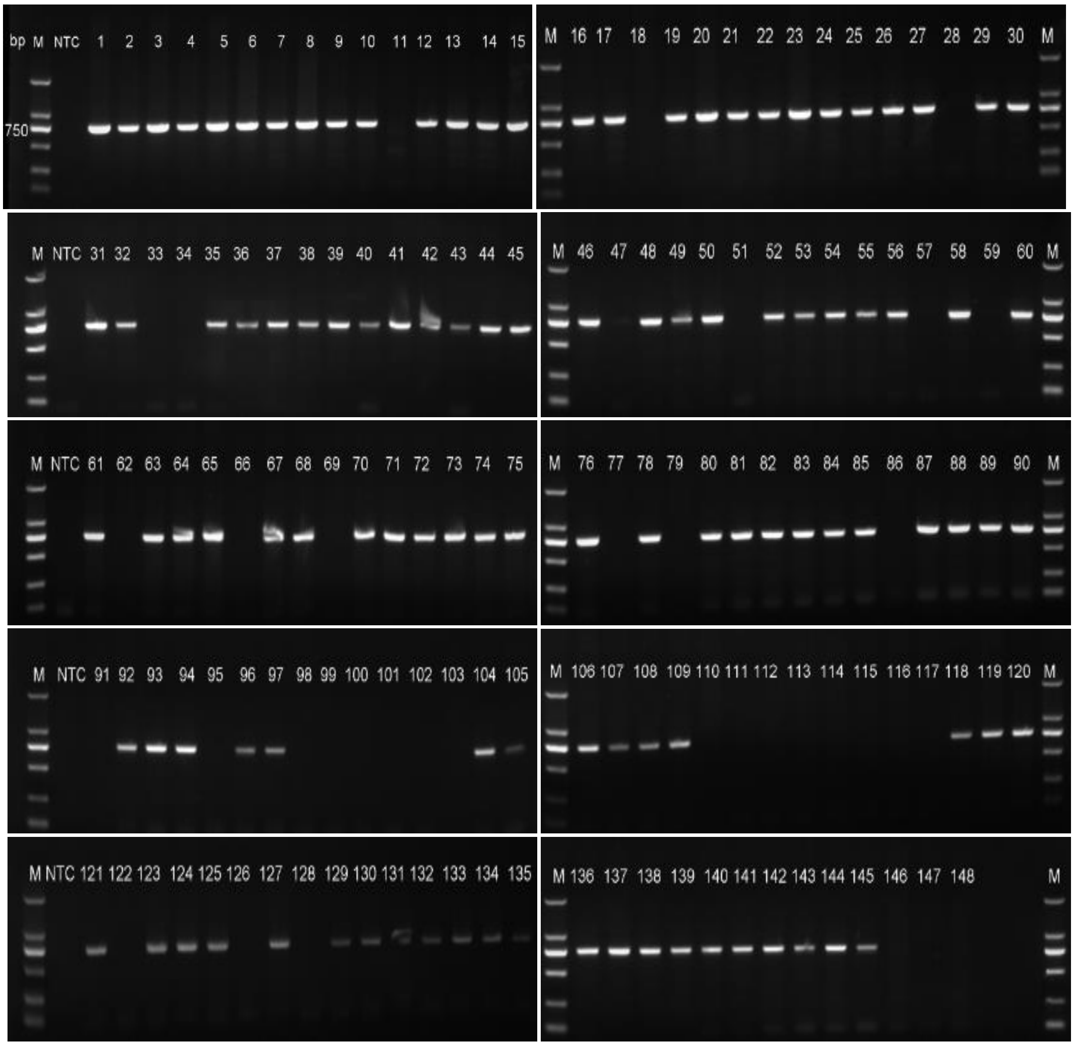

Supplementary Figure 15. PCR analysis of the RedTE-based marker in 148 accessions. Source data are provided in Source Data file 1.

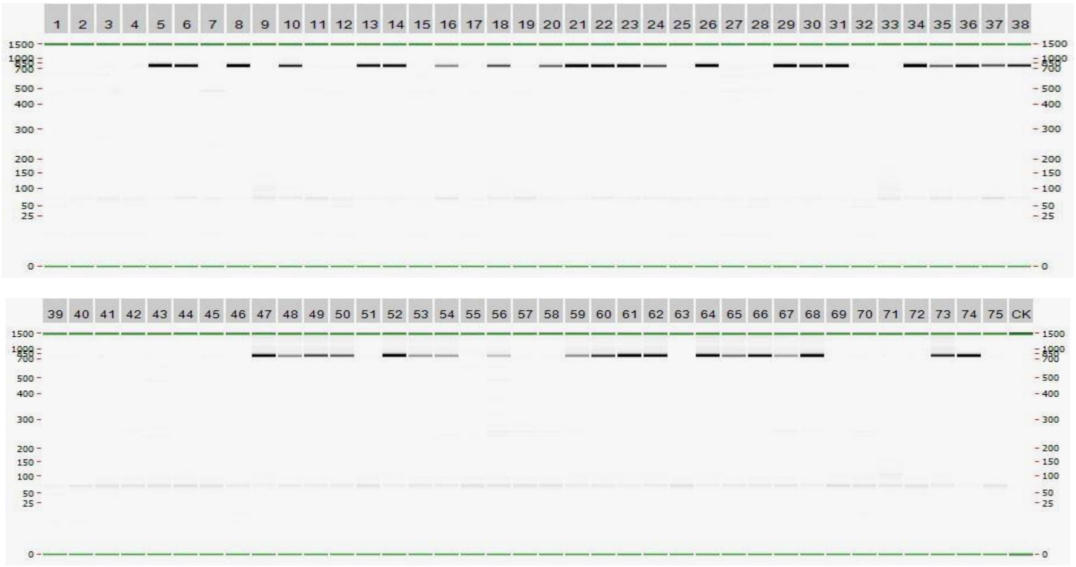

Supplementary Figure 16. Virtual gel-like photographs obtained from PCR analysis of the RedTE-based marker in 75 progenies of the cross of the Huayue (non red skin) and Honeycrisp (red skin) using a LabChip GX Touch instrument (1k-chip). Source data are provided in Source Data file 1.

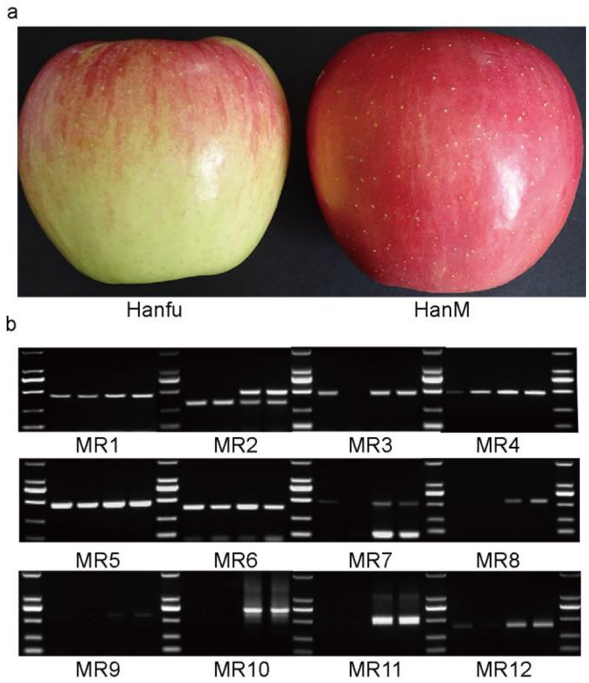

Supplementary Figure 17. DNA methylation status of redTE and the promoter region of *MdMYB1*. **(a)** Phenotypes of different red-skinned fruits of Hanfu (left) and its red sport HanM (right) at the ripening stage. Under the same condition, when the Hanfu fruits begins to colour, the HanM fruits has turned fully red. **(b)** McrBC-PCR identification DNA methylation levels of the promoter region of *MdMYB1* (MR1-MR7) and redTE transposon (MR8-MR12) between HanM and Hanfu. Lane1, 2, 3 and 4 indcated McrBC-treated DNA of HanM, McrBC-treated DNA of Hanfu, DNA of HanM without McrBC-treated, and DNA of Hanfu without McrBC-treated, respectively. Source data are provided in Source Data file 1.

Supplementary Table 1. Sequencing data sets generated in this study

	Sample	Tissue	Read type	Average Reads Length (bp)	Bases (bp)	Base coverage
Genome data	HFTH1	young leaf	Illumina, paired-end	2 X 150	43,339,156,500	65.62
	HFTH1	young leaf	Illumina, paired-end (Hi-C libraries)	2 X 100	95,490,716,800	144.57
	HFTH1	young leaf	PacBio, single-end	13,107	77,022,456,727	116.61
	HFTH1	young leaf	Bionano	178,908	147,797,940,000	223.77
	HFP	young leaf	Illumina, paired-end	2 X 150	50,752,481,700	76.84
	**Sample**	**Tissue**	**Read Type**	**Average Reads Length (bp)**	**Bases (bp)**	**Percent of Mapped**
RNA-seq data	HFP	fruit (break stage)	Illumina, paired-end	2 X 100	19,877,666,800	95.84%
	HFP	fruit (red stage)	Illumina, paired-end	2 X 100	19,846,611,600	95.57%
	HFTH1	young leaf	Illumina, paired-end	2 X 150	6,199,491,000	95.37%
	HFP	young leaf	Illumina, paired-end	2 X 150	6,232,787,400	93.60%
	HFTH1	mature leaf	Illumina, paired-end	2 X 150	6,206,832,000	94.86%
	HFP	mature leaf	Illumina, paired-end	2 X 150	6,167,440,200	93.20%
	HFP	branch	Illumina, paired-end	2 X 150	6,222,601,800	93.36%
	HFTH1	branch	Illumina, paired-end	2 X 150	6,421,635,900	95.32%
	HFP	flower (Early flowering period)	Illumina, paired-end	2 X 150	6,200,427,600	93.03%
	HFP	flower (Full bloom period)	Illumina, paired-end	2 X 150	6,166,718,700	92.99%

Supplementary Table 2. Assembly statistics of the HFTH1 genome

Assembly	Contigs			Scaffolds		
	N50 (kb)	No. of N50	Total size (Mb)	N50 (kb)	No. of N50	Total size (Mb)
Pacbio[a]	4,628.41	48	656.52	-	-	-
Optical Map	-	-	-	689.60	309	683.25
PacBio + Optical Map	4,625.47	49	656.52	10,176.19	21	658.81
PacBio + Optical Map + Gap-closure[a,b]	7,085.53	31	658.90	11,517.32	20	660.26
PacBio + Optical Map + Gap-closure + Hi-C[c]	6,988.12	32	658.90	37,138.69	8	660.50

[a]Including two steps polishing strategies using PacBio long reads and Illumina short reads.

[b]After removing the two organelle genomes.

[c]Three misassembled contigs were split according to the Hi-C map.

Supplementary Table 3. Comparison of genome features between the HFTH1 genome and other published genomes

	HFTH1	GDDH13	Li et al.	Velasco et al.	TAIR10	RGAP7
Total genome length (Mb)	660.50	643.20	632.42	603.90	119.67	374.47
Number of contigs	502	2,150	10,178	122,146	169	963
Contig N50 (kb)	6,988.12	620.00	104.48	13.40	10,898.02	7,711.35
Maximum contig length (kb)	18,010.64	2,368.17	958.42	NA	14,600.99	17,510.76
Complete BUSCOs of genome (%)	97.00	96.00	72.00	NA	98.20	98.30
TEs proportion (%)	59.78	57.96	NA	42.40	10.00	35.00
Annotated protein-coding genes	44,677	42,140	53,922	63,141	27,416	39,045
Complete BUSCOs of genes (%)	95.90	94.90	51.50	86.70	99.50	95.70

NA, not available.

Supplementary Table 4. Assessment of the quality of the HFTH1 genome with Illumina short reads

Ref.	Reads	Total Reads	Pair Mapped		Singletions Mapped		Proper-pair Mapped	
			Number	Percent	Number	Percent	Number	Percent
GDDH13	GDDH13	210,000,000	190,589,728	90.76%	2,689,424	1.28%	179,831,954	94.36%
HFTH1	HFTH1	140,000,000	135,358,604	96.68%	1,687,667	1.21%	133,611,170	98.71%
HFTH1	GDDH13	210,000,000	190,024,342	90.49%	2,894,939	1.38%	176,310,582	92.78%
GDDH13	HFTH1	140,000,000	134,201,132	95.86%	2,068,230	1.48%	125,152,002	93.26%

Supplementary Table 5. Assessment of the completeness of the apple genomes with ESTs

Ref	Dataset*	Number	Total Length(bp)	Sequence Covered by Assembly	Coverage rate > 90% in 1 Contig	Coverage rate > 50% in 1 Contig
Velasco *et al.*	All	326,941	158,164,334	98.14%	92.13%	97.30%
	>200	308,797	155,671,177	98.54%	93.22%	97.71%
	>1000	489	714,304	98.36%	69.93%	85.68%
Li *et al.*	All	326,941	158,164,334	96.65%	89.79%	95.67%
	>200	308,797	155,671,177	97.14%	90.90%	96.16%
	>1000	489	714,304	97.75%	69.32%	84.45%
GDDH13	All	326,941	158,164,334	97.84%	91.61%	96.71%
	>200	308,797	155,671,177	98.28%	92.73%	97.15%
	>1000	489	714,304	98.36%	71.77%	85.48%
HFTH1	All	326,941	158,164,334	98.02%	92.54%	97.10%
	>200	308,797	155,671,177	98.43%	93.67%	97.53%
	>1000	489	714,304	98.36%	71.77%	85.27%

*The ESTdata of apple was downloaded from NCBI.

Supplementary Table 6. Comparisons of repeat elements identified in the HFTH1 and GDDH13 genomes

Type		HFTH1			GDDH13		
		Numbers*	Length (bp)	Percentage (%)	Numbers*	Length (bp)	Percentage (%)
LTR	Total	263,476	304,090,586	46.15	263,056	274,776,584	43.97
	Copia	64,847	104,478,922	15.86	61,989	90,119,835	14.42
	Gypsy	97,084	167,415,052	25.41	98,799	153,511,663	24.57
	Caulimovirus	1,889	3,105,466	0.47	1,719	3,033,388	0.49
	Cassandra	5,714	2,474,135	0.38	6,013	2,390,885	0.38
	Caulimoviru	747	480,228	0.07	732	456,464	0.07
	Bel-Pao	66	559,592	0.08	52	461,201	0.07
	Other	93,129	25,577,191	3.88	93,752	24,803,148	3.97
DNA		219,477	64,831,994	9.84	223,351	65,448,607	10.47
Simple repeat		75,968	12,377,737	1.88	72,341	11,152,163	1.78
RC		13,958	3,510,647	0.53	11,508	3,311,031	0.53
SINE		4,534	747,268	0.11	5,519	752,401	0.12
LINE		27,725	30,357,558	4.61	24,808	27,737,398	4.44
Other		79921	13,423,085	1.99	85,255	13,841,797	2.22
Total		685,059	416,211,702	63.17	685,838	384,565,646	61.55

*most repeats fragmented by insertions or deletions have been counted as one element.

Supplementary Table 7. Part of the putative heterochromatin regions on the HFTH1 chromosomes

Chr	Start	End	Percent of Repeats	Length of Chr. (bp)
Chr01	3,750,000	5,749,999	91.92%	32,944,118
Chr02	23,000,000	25,999,999	92.17%	38,449,405
Chr03	19,500,000	20,499,999	90.82%	37,138,690
Chr04	13,000,000	14,749,999	91.21%	31,012,745
Chr05	14,250,000	15,499,999	91.24%	47,891,858
Chr06	12,000,000	16,249,999	91.23%	35,567,198
Chr07	15,000,000	16,499,999	91.13%	35,934,761
Chr08	16,750,000	18,249,999	91.72%	31,511,015
Chr09	21,500,000	23,499,999	92.70%	34,800,404
Chr10	13,500,000	15,249,999	92.19%	43,815,736
Chr11	21,500,000	22,749,999	92.00%	42,456,296
Chr12	12,750,000	13,999,999	91.36%	32,285,079
Chr13	28,750,000	30,999,999	91.91%	44,866,511
Chr14	12,250,000	13,249,999	95.58%	31,515,206
Chr15	38,750,000	41,249,999	91.87%	56,644,392
Chr16	29,500,000	30,499,999	91.42%	41,670,059
Chr17	18,000,000	19,499,999	91.87%	33,998,825

Supplementary Table 8. Enriched InterPro domains for genes with non-synonymous SNPs

InterPro ID	InterPro domain	Pvalue	Adjusted pvalue (FDR)
IPR002182	Domain NB-ARC	4.06E-13	1.53E-09
IPR000719	Domain Protein kinase domain	4.03E-08	7.61E-05
IPR008808	Domain Powdery mildew resistance protein, RPW8 domain	1.21E-06	1.53E-03
IPR001810	Domain F-box domain	1.48E-05	1.00E-02

Supplementary Table 9. Enriched InterPro domains for genes associated with GDSVs

InterPro ID	InterPro domain	P-value	Adjusted pvalue (FDR)
IPR000157	Domain Toll/interleukin-1 receptor homology (TIR) domain	5.38E-25	1.81E-22
IPR011713	Repeat Leucine-rich repeat 3	2.85E-09	6.41E-07
IPR003591	Repeat Leucine-rich repeat, typical subtype	9.34E-09	1.57E-06
IPR026961	Domain PGG domain	3.75E-05	5.05E-03
IPR013187	Domain F-box associated domain, type 3	6.04E-05	6.79E-03
IPR009737	Family Thioredoxin-like ferredoxin	7.23E-05	6.97E-03
IPR001611	Repeat Leucine-rich repeat	9.78E-05	8.24E-03
IPR013894	Domain RecQ mediated genome instability protein, N-terminal	1.20E-04	9.02E-03
IPR000454	Family ATP synthase, F0 complex, subunit C	3.92E-04	2.64E-02

Supplementary Table 10. Comparisons of the nucleotide differences of *MdMYB1*

No.	Position (Chr.09)	Base in the HFTH1 genome	Base in the GDDH13 genome	Data* used for ruling out the associations of SNPs and indels with fruit skin color
1	31,753,392	T	C	SRR3571235, SRR3571267
2	31,753,311	G	A	AB557640, AB557638, DQ886416, DQ886414, DQ886415, EU518249, HQ259417, KX822763
3	31,753,264	G	A	AB557640, AB557638, DQ886416, DQ886414, DQ886415, EU518249, HQ259417, KX822763
4	31,753,223	C	-	AB557640, AB557638, DQ886416, DQ886414, DQ886415, EU518249, HQ259417, KX822763
5	31,753,172	T	C	SRR3571267, SRR3571124, SRR3571143
6	31,753,143	A	G	SRR3571235, SRR3571267
7	31,753,094	G	C	AB557640, AB557638, DQ886416, DQ886414, DQ886415, EU518249, HQ259417, KX822763
8	31,753,075	C	T	AB557640,AB557638,DQ886416,DQ886414,DQ886415 ,EU518249,HQ259417 ,KX822763
9	31,752,948	G	A	Granny_Smith
10	31,752,094	G	A	Granny_Smith
11	31,752,086	G	A	AB557640, AB557638, DQ886416, DQ886414, DQ886415, EU518249, HQ259417, KX822763
12	31,752,073	C	A	SRR3571185, SRR3571235
13	31,752,043	A	T	AB557640, AB557638, DQ886416, DQ886414, DQ886415, EU518249, HQ259417, KX822763
14	31,752,020	T	C	SRR3571267, SRR3571243
15	31,751,924	T	-	SRR3571267, SRR3571235
16	31,751,919	T	A	SRR3571267, SRR3571235
17	31,751,009	G	A	AB557640, AB557638, DQ886416, DQ886414, DQ886415, EU518249, HQ259417, KX822763
18	31,750,919	TT	-	AB557640, AB557638, DQ886416, DQ886414, DQ886415, EU518249, HQ259417, KX822763
19	31,748,634	T	C	AB557640, AB557638, DQ886416, DQ886414, DQ886415, EU518249, HQ259417, KX822763

*Data downloaded from NCBI (accession number) or GDR (ftp.bioinfo.wsu.edu).

Supplementary Table 11. Resequencing data from the cultivars at GDR*

Skin color	cultivars
red	Braeburn, Coxs Orange Pippin, Fuji, Red Delicious, Red Dougherty, McIntosh Summerland.
Non-red	Golden Delicious, Goldrush, Granny Smith.

* Data downloaded from ftp.bioinfo.wsu.edu.

Supplementary Table 12. Primer sequences used for McrBC-PCR analysis

Gene name	Primer sequence (5'-3')	location from ATG of MdMYB1
MR1	GGTATCTTATGGTGGTCAAAGATG	-440 to -3
	CTTATCTGCTAGCAGCTAAGCTTA	
MR2	CTGAGATTGACTCTTGTGAAAGCT	-856 to -383
	GTGAATGCAGAATCGTGTAACTAGT	
MR3	TTAACGGAATCCAACGAAGACAAG	-1246 to -780
	GCTACACCTAACACATTGCTCAAT	
MR4	TGAGATAGGTCCGGTTCTATTTCT	-1657 to -1184
	ATCCCTTTCCCTTATTTGTTCCGT	
MR5	CAATTGCAGTGCTCAGAAATCGTT	-2044 to -1590
	CCGGACCCGTTTATAAATAGAACA	
MR6	CCATTTCCACCGTTCATTTCTAAG	-2255 to -1872
	AACAGCAAACACCCAAAATCCCTT	
MR7	CACTAGCTTCGGATTCCTTAGGA	-2978 to - 2501
	CGGTTTAGTTTCTGGGAATTCACA	
MR8	GGATTGTTCCTGCTGTCTCTCTGT	-3515 to -2957
	CCTAAGGAATCCGAAGCTAGTGAACT	
MR9	GTGCTGTTGATGCCAACGGAAGAT	-4172 to -3488
	GTCAACAGAGAGACAGCAGGAACAA	
MR10	GCATCTCTCTATGGCCACGAAGAAC	-4749 to -4000
	TGCGCAAACTCTCACAACTTGAGATC	
MR11	CTCCATATCGGCTTTATTGCCAAT	-5198 to -4725
	GTTCTTCGTGGCCATAGAGAGAT	
MR12	CCAAAATTTAGTAGCGAAAACAACTTTTC	-5594 To -5178
	GCCGATATGGAGAAATCCTGTTTGGCT	

Supplementary Table 13. Probe sequences used for EMSA analysis

Probe name	Probe sequence (5'-3')
CBF2BiotinF	CTATCTATGCCGACTTAAGATATCA
CBF2BiotinR	TGATATCTTAAGTCGGCATAGATAG
CBF2CF	CTATCTATGCCGACTTAAGATATCA
CBF2CR	TGATATCTTAAGTCGGCATAGATAG
CBF2BiotinMF	CTATCTATGTTATTGGAAGATATCA
CBF2BiotinMR	TGATATCTTCCAATAACATAGATAG
