# Supplementary material for: A high-quality apple genome assembly reveals the association of a retrotransposon and red fruit colour
Source: Nat Commun. 2019 Apr 2;10:1494. doi: 10.1038/s41467-019-09518-x (PMC6445120; doi:10.1038/s41467-019-09518-x)
Supplement: Supplementary file 3 — Reporting Summary [file 41467_2019_9518_MOESM3_ESM.pdf]

nature research

Corresponding author(s): Peihua Cong

Last updated by author(s): Jan 31, 2019

Reporting Summary

Nature Research wishes to improve the reproducibility of the work that we publish. This form provides structure for consistency and transparency in reporting. For further information on Nature Research policies, see Authors & Referees and the Editorial Policy Checklist.

Statistics

For all statistical analyses, confirm that the following items are present in the figure legend, table legend, main text, or Methods section.

n/a	Confirmed	
☒	☐	The exact sample size (*n*) for each experimental group/condition, given as a discrete number and unit of measurement
☒	☐	A statement on whether measurements were taken from distinct samples or whether the same sample was measured repeatedly
☒	☐	The statistical test(s) used AND whether they are one- or two-sided<br>*Only common tests should be described solely by name; describe more complex techniques in the Methods section.*
☒	☐	A description of all covariates tested
☒	☐	A description of any assumptions or corrections, such as tests of normality and adjustment for multiple comparisons
☒	☐	A full description of the statistical parameters including central tendency (e.g. means) or other basic estimates (e.g. regression coefficient) AND variation (e.g. standard deviation) or associated estimates of uncertainty (e.g. confidence intervals)
☒	☐	For null hypothesis testing, the test statistic (e.g. *F*, *t*, *r*) with confidence intervals, effect sizes, degrees of freedom and *P* value noted<br>*Give P values as exact values whenever suitable.*
☒	☐	For Bayesian analysis, information on the choice of priors and Markov chain Monte Carlo settings
☒	☐	For hierarchical and complex designs, identification of the appropriate level for tests and full reporting of outcomes
☒	☐	Estimates of effect sizes (e.g. Cohen's *d*, Pearson's *r*), indicating how they were calculated

Our web collection on statistics for biologists contains articles on many of the points above.

Software and code

Policy information about availability of computer code

Data collection	Flow cytometry data were collected and analysed using Flomax (v2.82) software.
Data analysis	Falcon (v0.4), Quiver, Pilon (v1.20), IrysView, PBSuite (v14.9.9), BLASR, bowtie2, Lachesis, Canu (v1.3), Tandem Repeats Finder (v4.09), LTR_FINDER (v1.0.6), RepeatModeler (v1.0.5), RepeatMasker (v4.0.6),MAKER (v2.31.8), BUSCO software (v3.0.1), Exonerate (v2.2.0), Histat2 (v2.05), StringTie (v1.3.0),SNAP (V2006-07-28), Augustus (v3.2.2), InterProScan (v5.24), StringTie, BEDTOOLS (v2.23.0), OrthoMCL (v2.0.9), Mafft (v7.058), PAL2NAL (v14), Gblocks (v0.91b), RaxML (v8.0.19), PAML package (v4.6), SynMap, Genome Analysis Toolkit (GATK, v3.8), Sniffles (v1.0.7), MUMmer (v3.07), SAMTOOLS (v1.2), LTR_retriever, LTRharvest, LTR_FINDER, R, Python. Perl. Specific parameters used during run-time are provided in the methods. All softwares or scripts are available from official websites or GitHub as indicated in the methods.

For manuscripts utilizing custom algorithms or software that are central to the research but not yet described in published literature, software must be made available to editors/reviewers. We strongly encourage code deposition in a community repository (e.g. GitHub). See the Nature Research guidelines for submitting code & software for further information.

Data

Policy information about availability of data

All manuscripts must include a data availability statement. This statement should provide the following information, where applicable:
- Accession codes, unique identifiers, or web links for publicly available datasets
- A list of figures that have associated raw data
- A description of any restrictions on data availability

Data generated during the study are deposited in the NCBI under study PRJNA482033. Raw data (PacBio and Illumina reads) have been deposited in the Sequence Read Archive (SRA) under study accession number SRX4557792, SRX4557793, SRX4557794. RNA-seq data of ten tested samples from 'Hanfu' are available under the SRA accession numbers SRX4557795-SRX4557802, SRX4557790 and SRX4557791. Genome assembly and annotation data has been deposited at GenBank under the

accession number RDQH00000000. FASTA files of chromosomes and genes, gff files for gene models also can be downloaded from https://github.com/moold/Genome-data-of-Hanfu-apple.

Field-specific reporting

Please select the one below that is the best fit for your research. If you are not sure, read the appropriate sections before making your selection.

☒ Life sciences ☐ Behavioural & social sciences ☐ Ecological, evolutionary & environmental sciences

For a reference copy of the document with all sections, see nature.com/documents/nr-reporting-summary-flat.pdf

Life sciences study design

All studies must disclose on these points even when the disclosure is negative.

Sample size	No sample size calculation was required for this study.
Data exclusions	No data exclusion
Replication	qRT-PCR and redTE marker vertification were repeated three times.
Randomization	here is not any randomized experimental group in our studies.
Blinding	No blinding was required for this study.

Reporting for specific materials, systems and methods

We require information from authors about some types of materials, experimental systems and methods used in many studies. Here, indicate whether each material, system or method listed is relevant to your study. If you are not sure if a list item applies to your research, read the appropriate section before selecting a response.

Materials & experimental systems

n/a	Involved in the study	
☒	☐	Antibodies
☒	☐	Eukaryotic cell lines
☒	☐	Palaeontology
☒	☐	Animals and other organisms
☒	☐	Human research participants
☒	☐	Clinical data

Methods

n/a	Involved in the study	
☒	☐	ChIP-seq
☐	☒	Flow cytometry
☒	☐	MRI-based neuroimaging

Flow Cytometry

Plots

Confirm that:

☒ The axis labels state the marker and fluorochrome used (e.g. CD4-FITC).

☒ The axis scales are clearly visible. Include numbers along axes only for bottom left plot of group (a 'group' is an analysis of identical markers).

☒ All plots are contour plots with outliers or pseudocolor plots.

☒ A numerical value for number of cells or percentage (with statistics) is provided.

Methodology

Sample preparation	Nuclei were isolated from young leaves of HFTH1 and Hanfu in spring ,using DAPI staining for 30 seconds.
Instrument	Partec CyFlow Space
Software	FACS data analyses were performed using Flomax (v2.82) software
Cell population abundance	5000 cells were collected for each sample. About ck sample 1#, the percentage of main peak was 53.88%, mean value of peak was 199.47, cv of peak was 7.15%. About sample mixture of 1 and 2,the percentage of main peaks were 24.72% and 32.79%, mean value of peaks were 200.03 and 310.15, cv of peaks were 5.49% and 3.59%. Total nuclei populations were gated using relative fluorescence intensity: the proportions of nuclei with different ploidy levels were determined based on their relative

fluorescence intensity: Hanfu is diploids (2N) as a reference, and the HFTH1 was calculated as a triploids (3N), according to peak position (Supplementary Figure 3).

Gating strategy	Total nuclei populations were gated using DAPI intensity. in DAPI+ singles cells,the proportions of nuclei with different ploidy levels were determined based ontheir DAPI intensity (Extended Supplementary Figure 3).

☒ Tick this box to confirm that a figure exemplifying the gating strategy is provided in the Supplementary Information.
