# Supplementary material for: A high-quality apple genome assembly reveals the association of a retrotransposon and red fruit colour
Source: Nat Commun. 2019 Apr 2;10:1494. doi: 10.1038/s41467-019-09518-x (PMC6445120; doi:10.1038/s41467-019-09518-x)
Supplement: Supplementary file 4 — Description of Additional Supplementary Files [file 41467_2019_9518_MOESM4_ESM.docx]

**Description of Additional Supplementary Files**

File Name: Supplementary Data 1
Description: Identification of RedTE markers for Malus accessions used in this study.

File Name: Supplementary Data 2
Description: Seventy-five progenies of the cross of Huayue (non red skin) and ‘Honeycrisp’ (red skin).
