# Supplementary material for: A high-quality apple genome assembly reveals the association of a retrotransposon and red fruit colour
Source: Nat Commun. 2019 Apr 2;10:1494. doi: 10.1038/s41467-019-09518-x (PMC6445120; doi:10.1038/s41467-019-09518-x)
Supplement: Supplementary file 2 — Peer Review File [file 41467_2019_9518_MOESM2_ESM.pdf]

Reviewers' comments:

Reviewer #1 (Remarks to the Author):

In this manuscript Zhang and colleagues present a high-quality apple genome assembly for the Hanfu variety, which is an important cultivar in China.
In order to detect structural variations between the Hanfu and Golden Delicious genomes, the authors then compare these two high-quality genomes.
Notably, overall the two genomes are quite similar showing a high co-linearity and very similar genome size.
The authors find that rearrangements and indels are often associated to transposable element mobility. They then focus on one specific TE (redTE), that is present over 3kb upstream of the MdMYB1 (MdMYB10) gene in the Hanfu but not in the Golden Delicious variety. Because this TE is only present in red apples, the authors conclude that redTE is directly responsible for the red skin colour in all tested apple varieties.

General comments
The manuscript is well written, clear and straight-forward. The genome seems to be of excellent quality.
To be able to verify the authors claims I would have liked to have access to the genome.
Unfortunately I could not find/access the NCBI project mentioned in the manuscript.
It is very important that all fasta, gff3 and other files will be made available to the community.

The redTE aspect of the manuscript is very interesting but needs to be strengthened: The authors show a very clear correlation about the presence of redTE and fruit color, but they do not demonstrate that redTE is truly responsible for the anthocyanin accumulation in the apple fruit skin upon high light exposure.
While it would be really nice to carry out a CRISPR-Cas9 knock out of redTE to see how that would affect fruit colour, this would be far too time consuming (maybe for future projects?). At least the authors should demonstrate that redTE shows a similar transcriptional regulation as MdMYB1 upon high light exposure. This should be the case since redTE has two LTRs, one presumably driving MdMYB1 and the other redTE itself.

Unfortunately, the DNA methylation changes observed that may explain the changes on fruit colour between Hanfu and HanM do not fall within redTE, which may contradict the hypothesis that redTE is necessary for the red fruit phenotype. The authors should discuss this point and compare their data with the studies that have previously been published (El-Sharkawy et al and Telias et al).

Detailed comments:
Typo in suppl. Fig. 1
"Embroy" instead of Embryo

Supplementary Figure 3: Define HFP

Does Copia-7 correspond to the HODOR repeat that was identified in GDDH13? It shows a very similar chromosomal distribution. If it is the same it should be named as such.

line 189: " 'Hanfu' evolved much faster, providing resistance to various environmental stresses."
The authors cannot state this as the opposite (that Golden rapidly lost resistance) may as well be true.

line 212: "after ‘Hanfu’ diverged from ‘Golden Delicious’"
I guess the authors mean after Hanfu and GD diverged from a common ancestor?

line 273: "we found that approximately 2.9% of the LTR-RTs in the HFTH1 genome may have been deleted and replaced with completely unrelated sequences in the GDDH13 genome (Fig. 3b)."
It is unclear to me how the authors get to this conclusions. Are there remnants of the TEs, the actual TSD of redTE?

paragraph at line 325: There is also the epigenetic silencing of MdMYB1/10 that has been shown previously by multiple reports. Do these lines also carry the redTE? Are the DNA methylation changes associated with the redTE? Are the MR primers the same as the ones used in the other studies?

paragragh at line 336: this paragraph is too hypothetical and some additional experimental data should be provided (see previous comments).

What are the redTE copy number variations between Golden Delicious and Hanfu? Maybe for all TEs?

The NCBI database does not contain an entry for PRJNA482033

Figure 4: positive control missing!

Reviewer #2 (Remarks to the Author):

This study presents a high-quality apple genome with a better contiguity and completeness than previously sequenced apple genomes. The availability of genome sequence from another apple genotype is certainly helpful to the community as the genetic variation can be identified and exploited for apple breeding. However, the manuscript is largely descriptive and does not provide much novel information on apple genome evolution and biology. In addition, most of the conclusions the authors drew are not supported by the data they presented (details below).

Genome assembly, annotation and assessment
Line 106-107: The authors need to provide a genome size estimation of HFTH1 by kmer and/or flow cytometry analysis. Genome sizes estimated for apple in different studies are highly inconsistent, e.g., 742 Mb in Velasco et al (2010), 651Mb in Daccord et al. (2017), and 743-796 Mb in https://link.springer.com/article/10.1007/BF02672069.

Line 108-109: I agree that the assembly of HFTH1 represents a notable improvement over Velasco et al (2010) and Li et al (2016), but I think the improvement over the GDDH13 assembly seems incremental.

Lines 150-157: The method to assign centromeres is questionable. The authors may use the algorithm described in https://www.ncbi.nlm.nih.gov/pubmed/23363705 for contromere identification. The conclusion on the evolutionary association of a TE family and centromere based on the presented analysis is most likely speculative.

Line 158: Unlike human, gorilla and goat, there could be much more variations between genomes of different apple varieties. To confirm gaps of GDDH13 filled by the HFTH1 genome are real gaps in the GDDH13 assembly instead of indels between these two genomes, the authors should align GDDH13 reads to the filled gaps to check the coverage. In addition, could the GDDH13 genome be used to fill gaps in the HFTH1 genome?

Genome comparison

Lines 183 and 572-574: First, why Velasco et al was not included to identify shared SNPs? Second, why only shared SNPs were used in the analysis? Many real SNPs that were captured by only one assembly would be missed.

Line 184-185, "Approximately 3.45% of non-synonymous SNPs were located within 36.20% of annotated genes": This sentence seems totally wrong. All non-synonymous SNPs should be located in coding regions. Not sure why only 3.45% here? What does "annotated genes" mean? All predicted genes in the genome or only those with functional annotations?

Line 185-190: Why only top 100 genes were used for enrichment analysis? This does not make any sense. All genes with non-synonymous SNPs should be used. How the authors conclude that "these genes ontologies in 'Hanfu' evolved much faster, providing resistance to various environmental stresses" from this enrichment analysis? I couldn't see any evidence here.

Line 192: "(including presence variations) that were with > 100 bp in length" should be "(including presence/absence variations) that were > 100 bp in length".

Line 194: Based on Fig. 2a, unlike SNPs, the PAV analysis did include the genome of Velasco et al. Why use two different criteria for SNP and PAV analysis? In addition, Fig.2a is very hard to understand. They are not venn diagrams. For example: is "3,287" the number of shared PAVs between Velasco et al and GDDH13 or specific to Velasco et al?

Line 199-202: These two sentences are disconnected. What is the logic to put together "the deletion in 5' UTR of MdCBF2" and "MdCBF2 and other MdCBFs were responsive to low-temperature treatment"?

Line 220-222: The conclusion here is too speculative. The low diversity in one end of chromosome 5 could be due to introgression and fixation during domestication and breeding process, instead of long-term evolution after WGD.

Dynamic evolution of LTR retrotransposons

The evolution of TEs in apple has been emphasized previously in Daccord et al (2017), and the comparative analyses here in different apple accessions are mostly descriptive and relevant conclusions are likely speculative.

Line 239-242: These statements need more evidence, otherwise it's too speculative.

Line 246: "ten tested tissues" should be "ten tested samples".

Line 253: The proportion of the gene regions plus 5kb flanking regions versus the whole genome should be shown here, otherwise 62.05% may not be a significant value.

Lines 259-260: Fig S14a (box plots) shows the expression as Log (No. of mapped reads). Normalization is needed here. In addition, the statement "indicating that most specific LTR-RTs…gradually weakened" is completely speculative and is not even supported by the data, as in Fig S14a, TE expression is not significantly different in all samples from HFTH1.

Line 262-270: Indel-associated mutations have been widely reported in plants and animals. This part just confirms findings from other studies without providing much novel information.

Lines 276-277, "deletion events might have made a greater impact than insertions did during the evolution of TEs": deletions and insertions are relative depending on which genome is used as the reference. "deletions" in HFTH1 mean "insertions" in GDDH13. Therefore, this general conclusion is totally wrong.

Line 278-279: This statement is too speculative with very weak evidence support.

Retrotransposon as an enhancer of MdMYB1

The role of MYB1 in regulating anthocyanin biosynthesis has been reported in apple and other plants, and transcriptional regulation of MYB1 by transposon insertion has also been report in other plants (e.g., https://www.ncbi.nlm.nih.gov/pubmed/15143274; https://www.ncbi.nlm.nih.gov/pubmed/20855520); therefore, data presented here, despite being interesting to some degree, mainly confirms similar mechanisms found in other plants. However, my major concern here is that most conclusions are lack of support with strong evidence.

Line 292-293: First, I could not find this conclusion from ref 15. Second, if this is the case, have you check the MdNYB1 allele in HFTH1? A multiple sequence alignment of different alleles should be provided? What if the MdMYB1 allele in HFTH1 contains additional base differences not found in the three reported alleles?

Line 297-310: The authors identified several SNPs and two large indels in the upstream of MdMYB1. How they eliminate the possibility that these SNPs and the other indel could play roles in regulating MdMYB1? I couldn't find the related information from the manuscript. The authors abruptly pick the long insertion for analysis without any reasoning.

Line 314-315, "redTE was not found in the non red-skinned accessions": I do find in Table S10 two non-red-skinned accessions ("Roxbury Russet" and "Ningguan") containing redTE.

Line 322-324: I couldn't follow the logic here. Why talk about flesh color here? It's OK to conclude that red-flesh in these three accessions is independent of redTE, but the conclusion that it is caused by constitute MdMYB10 expression is highly speculative.

Line 331-335, Figure S15: I am confused here. Why 'Hanfu' (red stripe) and 'HanM' (fully red) were used here for the analysis? The region MR8-MR11 corresponds to redTE, which was heavily methylated in both 'Hanfu' and 'HanM'. Then how they conclude methylation could be associated with red phenotype? Did the author mean the methylation changes of MR3 and MR7 were induced by redTE? If this is the case, then I did not see any evidence provided.

Line 336-343: This is purely computational prediction without any experiment evidence supporting the conclusion made here.

Line 360-373: Ref 34 describes the identification of a TE using differential display between Jonagold and its color mutants. Authors from this paper never concluded that "red apples have arisen from non red-skinned apples by transposon-induced mutation". Discussion in the section is highly speculative and I couldn't find any of their data to support their speculations. For example, I could not find expression data to support redTE as "a tissue-specific enhancer".

Lines 382-383: Such strong conclusion can only be made if the full "redTE" sequences are available for all mentioned apple accessions.

Line 384-387: As described in Ref 6, M. sieversii in Xinjiang has not been involved in the domestication process. Therefore, this statement is not correct.

Line 406-411: Another speculation. A lot of new data need to be generated to support the TE-regulated expression of MdACS3 and the usefulness of the said marker.

Reviewer #3 (Remarks to the Author):

In the manuscript entitled “A high-quality apple genome assembly reveals a retrotransposon controlling red fruit colour” by Zhang et al the authors present a large data set further refining the apple genome, from its first published draft, to a more highly resolved resource. They then focus on one trait - skin color - to illustrate the versatility of having this new detail.
The final assembly has very impressive statistics - similar or better than model plant genomes.

The paper is well written and the data is convincing.
I have a few issues with the way the paper introduces the work. Firstly - it wasn’t until the first section of the results, that I realised the genome wasn’t a double haploid or some other trick of homozygosity. Somehow the authors should state clearly earlier what was done to make HFTH1, and why it lacks much of the heterozygosity that apple usually has.

Line 65 - the genetic basis of apple skin color is well understood - perhaps not the mutation that drives yellow verses green skin.

Line 91 - why was HFTH1 more homozygous?

Line 228-246 - the difference between LTR-RTs in pear (40) and apple (7800) is very impressive - are the authors certain? Could there have been some masking done in the pear assembly?

Discussion - some mention of the Ruby gene in citrus would be good - there is a transposon that drives expression of the citrus MYB during the cold in orange - could expression out of the apple LTR-RT be cold induced?

The followings are the responds to the reviewers' comments:

Reviewer reports

Reviewer #1:

In this manuscript Zhang and colleagues present a high-quality apple genome assembly for the Hanfu variety, which is an important cultivar in China. In order to detect structural variations between the Hanfu and Golden Delicious genomes, the authors then compare these two high-quality genomes. Notably, overall the two genomes are quite similar showing a high co-linearity and very similar genome size. The authors find that rearrangements and indels are often associated to transposable element mobility. They then focus on one specific TE (redTE), that is present over 3kb upstream of the *MdMYB1 (MdMYB10)* gene in the Hanfu but not in the Golden Delicious variety. Because this TE is only present in red apples, the authors conclude that redTE is directly responsible for the red skin colour in all tested apple varieties.

Response: We thank the reviewer for giving us positive comments and some proposals in point.

General comments:

The manuscript is well written, clear and straightforward. The genome seems to be of excellent quality. To be able to verify the authors claims I would have liked to have access to the genome. Unfortunately I could not find/access the NCBI project mentioned in the manuscript. It is very important that all fasta, gff3 and other files will be made available to the community.

Response: we have asked NCBI to release all data of this project. FASTA files of chromosomes and genes, gff files for gene models also can be downloaded from https://github.com/moold/Genome-data-of-Hanfu-apple.

The redTE aspect of the manuscript is very interesting but needs to be strengthened: The authors show a very clear correlation about the presence of redTE and fruit color, but they do not demonstrate that redTE is truly responsible for the anthocyanin accumulation in the apple fruit skin upon high light exposure. While it would be

really nice to carry out a CRISPR-Cas9 knock out of redTE to see how that would affect fruit colour, this would be far too time consuming (maybe for future projects?). At least the authors should demonstrate that redTE shows a similar transcriptional regulation as MdMYB1 upon high light exposure. This should be the case since redTE has two LTRs, one presumably driving MdMYB1 and the other redTE itself.

Response: We thank the reviewer for positive comments and suggestions. It is a very good idea to use CRISPR-Cas9 knockout of redTE to vertify its function. But, ideally, it will take about 3-5 years to observe the phenotype of fruit after CRISPR-Cas9 knockout of redTE in apple. A CRISPR-Cas9 knockout projects will be carried out in our following work.

In view of suggestion, we carried out a transcriptional expression analysis of redTE and *MdMYB1* upon high light exposure & dark condition (bagged fruits) by RNA-seq. There is a silght relationshps of transcriptional expression of redTE and MdMYB1 between samples from high light exposure & dark condition. The mining of transcriptome data showed redTE to be transcribed in flanking LTR (482bp, gray regions within LTR sequence of redTE, see following), but reads within gray regions matching with redTE (Chr.9) is identical with another LTR of TE (Chr.6). Therefore, we did not distinguish transcriptional reads from redTE or other TE. As for how LTRs drives *MdMYB1*, by providing either other transcription factor binding site or influencing the chromatin state in a non-sequence specific manner, or as non-coding RNA (eRNA), need to be further studied in future. So we didn't provide relative description with a transcriptional expression analysis of redTE and *MdMYB1* in our report.

Here, to demonstrate that redTE acts as an enhancer of *MdMYB1* expression, the construct with the redTE obviously increased expression of the luciferase reporter relative to the construct for the minimal 35S promoter alone. These results indicate that the redTE element acts as an enhancer of gene expression and may explain the increased expression of *MdMYB1*, which is similar to a transposable element providing an enhancer functioning as a long-distance enhancer of *tb1* gene expression in maize (Studer, Anthony, et al. "Identification of a functional transposon insertion in

the maize domestication gene tb1." Nature genetics 43.11 (2011): 1160.), see lines 353-361 and Figure 4d.

Additionally, we added to ananlysis of cross progenies: the 75 progenies analysis from the cross of the 'Huayue' (non red skin) and 'Honeycrisp' (red skin), also confirmed the perfect co-segregation of redTE and red phenotype. These results are now reported in the main text (lines 338-344), and a pedigree showing genotypes and phenotypes is shown in Supplementary Table 12, Fig. 4c and Supplementary Fig. 15.

TGTTGACCCTAGAAACTACAAAGCCTACGTGGCGCGCAGGCCGAATAATT
AATAAGCTAACTACGTCCTTCGGTGATTGCGGGGCGTGCCAACTCGTCGG
CCGAGGCTCGGCCGAGGAGTAAATTTGATGATGCTGCGTTGGGTCGCGCT
ACTGACTTCTGCGTCTTGCGATTGCGGCCGAGAAAGGAACGCGTCTCGGC
CTCTTGGGTTCTCGAGCCTGAAGACAAGGCTGCTATTTCTTACAAAGTTCA
CGAACCGAATTCGGCTTACAATGTGCCGAATGTAATAACTGTAACACCTC
ACCTCGCCGAGAAGGCTAATGAGATGACCTCGACCAACAAGGATTCGAA
AACCCTTCTCGACCGAGACTTGGATAGGCAATCGACCGTTCTCGCCGCAG
TGCTGTTGATGCCAACGGAAGATACTGCGAGACCGACCGACTCTACGGTG
ACAGAGCTATCTATGCCGACTTAAGATATCACCGGTTGCTTCCACAGTGCT
GTTGATGCCAACGGAAGATGTGTCAGCGAAAAAAGAAAAGAAAAAGATC
TCAAGTTGTGAGAGTTTGCGCAGGGCAATTTTGTATTGATGTTTGTGGGGC
CTTTTCTCTGTTGCTGAATGTCTTGTATTTATAGTAGCAGAACATCTGCTTG
TCGCGGTTGGATGATAAACATCTCAATCGCACCCAAAGTCTTTGTGACGG
AATATCCTGAGAAATAAAATCATGACCACATGTGAATCTGAAAAATAGGG
ACTTCTGCATGCCAATCCCGCTTTATCCAACTTTGACTTATCTCTATCCAA
ACACTGAATCTTTTGGGATGGTTCTACACGGCTCTTCACGTAAAAATATCA
CCTTGCTGTCATCCTGACAACCTAACCTTCCATCAAATTAGAAGCCTAGCT
AGGCTAGGCCTCTTTTTCTTGCCACATGCATGTTACGGCAGCCATCAATCA
AAATATATATATATATATATATATGTATATAATCATATATATATATAGCCG
AGTTGTTCTAACCCACACGGTACATTGTTAACAAATTCAAATTAAATCGG
ATTGTTCCTGCTGTCTCTCTGTTGACATATAATTTGAACCCTATTTATCTGG

TTTAAGAAGATGCATATAATTCGTATTAAAAGATAATTATGATAAAAACC
ATAATATTATCCTTTAACAAAAAATATCTTTCCTTTTCCATCCGACGGTTG
AGAACTATGCTTACTCAACGGCCTTGATTACACGGGCCGATGGTGTAAAT
TTGGGCCCAAACA

An LTR sequence of redTE

Unfortunately, the DNA methylation changes observed that may explain the changes on fruit colour between Hanfu and HanM do not fall within redTE, which may contradict the hypothesis that redTE is necessary for the red fruit phenotype. The authors should discuss this point and compare their data with the studies that have previously been published (El-Sharkawy et al. and Telias et al.).

Response: Thanks you for providing these insights. The DNA methylation changes, we compared our data with the studies that have previously been published. Indeed, this statement, “indicating that methylation may be associated with the red fruit phenotype”, is easy to misunderstand. We have revised and stated “indicating that redTE-induced epigenetic change may be associated with different coloration patterns in red-skinned apple”.

TE can alter epigenetic marks to nearby genes thereby modifying the host gene expression (Bucher, Etienne, Jon Reinders, and Marie Mirouze. "Epigenetic control of transposon transcription and mobility in Arabidopsis." Current opinion in plant biology 15.5 (2012): 503-510.). In fact, under reviewer’s guidance, we found that the degree of methylation in Hanfu is much higher than that of HanM (bud sports of Hanfu) in the region MR12 (Supplementary Fig. 16b), indicating that transposon-mediated epigenetic regulation may control the variable colour patterns, See lines 369-377. We have properly modified the statement as explained above.

Typo in suppl. Fig. 1 "Embroy" instead of Embryo

Response: This has been corrected as suggested (Fig. 1a), and we have moved suppl. Fig. 1 to Fig. 1a as other reviewer's suggestion.

Supplementary Figure 3: Define HFP

Response: We have added the definition of HFP (Supplementary Figure 2).

Does Copia-7 correspond to the HODOR repeat that was identified in GDDH13? It shows a very similar chromosomal distribution. If it is the same it should be named as such.

Response: Actually, Copia-7 shows a low similarity with HODOR and the HODOR repeat was identified as the most repetitive consensus sequence in the whole apple genome, but Copia-7 was identified as the most repetitive sequences in the identified heterochromatin regions (lines 153-163).

line 189: " 'Hanfu' evolved much faster, providing resistance to various environmental stresses."The authors cannot state this as the opposite (that Golden rapidly lost resistance) may as well be true.

Response: Thanks for the rigorous suggestion and this has been modified as "these genes in two cultivars may evolve under different selection pressures, providing resistance to various environmental stresses", see lines 197-199.

line 212: "after 'Hanfu' diverged from 'Golden Delicious'"

I guess the authors mean after Hanfu and GD diverged from a common ancestor?

Response: This has been corrected as suggested (lines 241-242).

line 273: "we found that approximately 2.9% of the LTR-RTs in the HFTH1 genome may have been deleted and replaced with completely unrelated sequences in the GDDH13 genome (Fig. 3b)."It is unclear to me how the authors get to this conclusions. Are there remnants of the TEs, the actual TSD of redTE?

Response: We have elaborated on this question (lines 281-287 and 668-671). The 'deletion' was defined in both cases: (1) if two TSDs exist at the corresponding

inserted site of the GDDH13 genome and the intervening sequence between two TSDs with length less than 100 bp or had no blast hits (BLASTN e-values ≤ 10) to the corresponding LTR-RT sequence in the HFTH1 genome. Partial sequence of this LTR-RT has been deleted or replaced with other TE sequences. (2) No TSDs exist at the corresponding inserted site of the GDDH13 genome. The LTR-RT with two flanking sequences has been deleted. Most of the "replaced sequences" are the remnants of other TEs, because these sequences cannot be aligned to the corresponding LTR-RT sequence in the HFTH1 genome, but can be aligned to other TEs at other positions in the GDDH13 genome. Two TSDs of redTE can be detected in the HFTH1 genome, but only one can be detected in the GDDH13 genome (Fig. 4a). In order to distinguish from 'deletion and insertion' events defined in previous SV analysis, we have modified the term 'deletion' in this section to 'excision'.

There is also the epigenetic silencing of MdMYB1/10 that has been shown previously by multiple reports. Do these lines also carry the redTE? Are the DNA methylation changes associated with the redTE? Are the MR primers the same as the ones used in the other studies?

Response: Because we could't get these special mutation lines that were used in other reports, we don't sure whether these special mutation lines also carry redTE. But we chose randomly the accessions of some bud sports harbor redTE in our study (Supplementary Table 10). Many studies have shown that TEs have adapted to supply the host genome with *cis* elements that can alter gene expression both genetically and epigenetically, thereby modifying the host gene expression (Lisch, Damon. "How important are transposons for plant evolution?" Nature Reviews Genetics 14.1 (2013): 49.), so the methylation changes may be induced by redTE in natural condition. But, further work needs to be performed to clarify this difficult problem, how redTE mediated genetic and epigenetic variation contributes to red phenotypic diversity and adaptation to light changes in apple in future.

A study of worldwide genetic diversity and pedigree records in apple estimated that

one yellow-skinned cultivar 'Golden Delicious (1916)' and four red-skinned cultivars ['Cox's Orange Pippin (1850)', 'Red Delicious (1880)', 'Jonathan (1826)' and 'McIntosh (1870)'] were the 'founders' of modern apple breeding. We cloned and sequenced the redTE of four red-skinned cultivars ['Cox's Orange Pippin', 'Red Delicious', 'Jonathan' and 'McIntosh'], and alignments sequence showed they were identical. Promoter of myb1 have a highly similarity in five cultivars. In general, it's ok that the MR primers are the same as the ones used in the other studies.

this paragraph is too hypothetical and some additional experimental data should be provided (see previous comments).

Response: This exactly is a result of computational prediction. According to your suggestion, we selected two transcription factor MdHY5, and MdCBF2 for further analysis. We tried to perform an experimental supplement by yeast one-hybrid to test whether MdHY5is able to bind the predicted elements of redTE, but there was self-activation when target fragments of redTE was fused to pAbAi for bait vector construction. Also, we tried to do it by EMSA, but GST-MdHY5 fusion protein was not detected in our prokaryotic expression system under different induction condition. Here, our EMSA result showed that MdCBF2 could bind the target motif of redTE, see lines 378-386 and Fig. 4e.

What are the redTE copy number variations between Golden Delicious and Hanfu? Maybe for all TEs?

Response: Thanks for nice suggestions, we have added the copy number variations between HFTH1 and GDDH13. See lines 321-326 for redTE copy number variations and Supplementary Table 6 for all TEs.

The NCBI database does not contain an entry for PRJNA482033

Response: we have asked NCBI to release all data of this project, FASTA files of

chromosomes and genes, gff files for gene models also can be downloaded from https://github.com/moold/Genome-data-of-Hanfu-apple.

Figure 4: positive control missing!

Response: Thanks for nice suggestions, we added the positive control using plasmid as template in Figure 4b. The plasmids, containing the target sequence, were confirmed by Sanger sequencing.

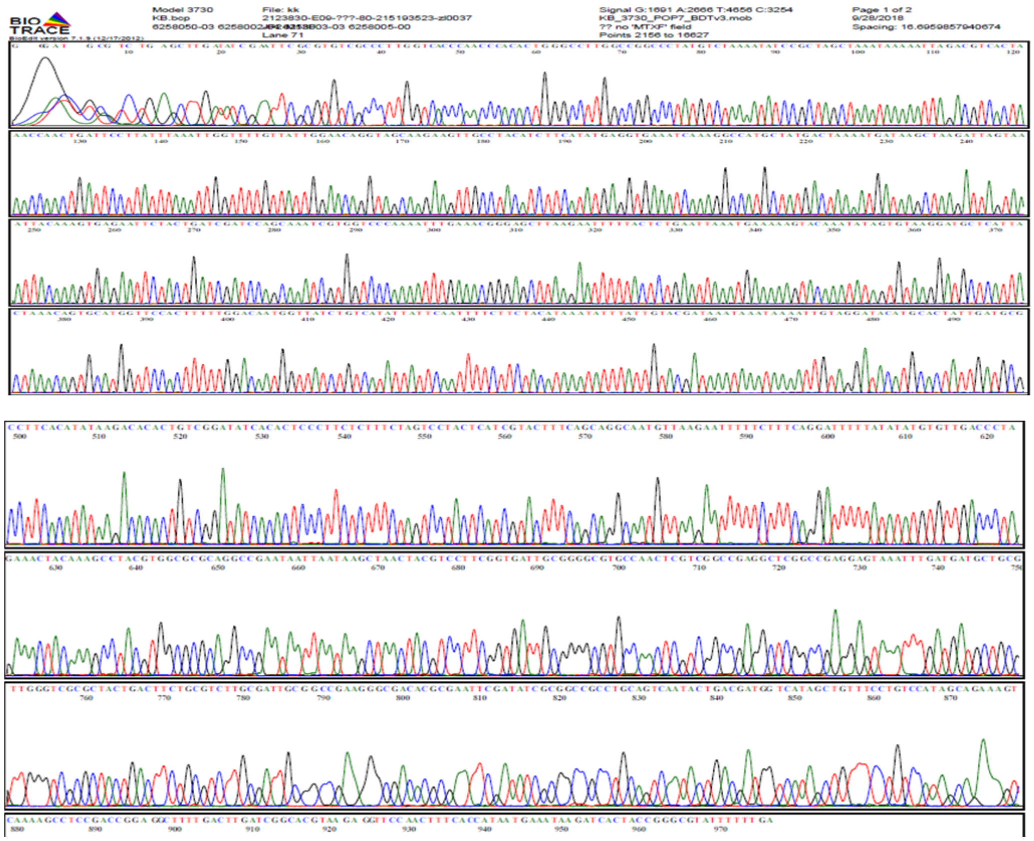

Reviewer #2 (Remarks to the Author):

This study presents a high-quality apple genome with a better contiguity and completeness than previously sequenced apple genomes. The availability of genome sequence from another apple genotype is certainly helpful to the community as the genetic variation can be identified and exploited for apple breeding. However, the manuscript is largely descriptive and does not provide much novel information on apple genome evolution and biology. In addition, most of the conclusions the authors

drew are not supported by the data they presented (details below).

Response: Thanks you for remarks and pointing out the shortcomings of our work. Here, we made a substantial improvement for our manuscript and polished it.

It was quite an achievement to assembly a novel apple genome with a better contiguity and completeness, which will provide much novel information on apple genome evolution and biology for the community. In our studies, we also found extensive genomic variations with far exceeding expectations, and especially the discovery of redTE is intriguing, which is an important step forward in understanding red colour in apple.

Genome assembly, annotation and assessment

The authors need to provide a genome size estimation of HFTH1 by kmer and flow cytometry analysis. Genome sizes estimated for apple in different studies are highly inconsistent, e.g., 742 Mb in Velasco et al (2010), 651Mb in Daccord et al. (2017), and 743-796 Mb in https://link.springer.com/article/10.1007/BF02672069.

Response: We have added more details about the genome size estimation of HFTH1 (lines 109-112). Its estimated genome size (708.54 Mb) is close to the estimated genome size (701 Mb) for apple by Li et al., but smaller than the genome size estimation in Velasco et al. (2010), larger than Daccord et al. (2017). The differences may be due to genotype, sequencing error and heterozygous rate, according to this paper (https://arxiv.org/ftp/arxiv/papers/1308/1308.2012.pdf). In our opinion, the predicted genome size of apple should be more than 650 Mb, because approximately 2.11% Illumina reads of HFTH1 could not be mapped to our assembly. Daccord et al. reported the genome size estimation of GDDH13 is only 651 Mb, but this may be underestimated because they used error corrected reads to perform genome size estimation.

In addition, we determined the genome size of HFTH1 and HANFU, and Golden Delicious as a reference by flow cytometry, based on the method of propidium iodide (PI) staining. The result showed that there were no significant differences in genome size among Golden Delicious, HANFU and HFTH1, according to the ratio of

fluorescent peak value of HANFU and HFTH1 to the Golden Delicious reference.

**Accessions**	**Fluorescent value**	**Ploidy**	**The haploid of fluorescent value**	**Genome size(Mb)**	**figure**
Golden Delicious	104.84	2n	52.42	701	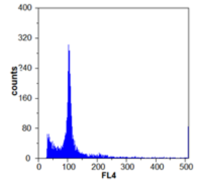
HANFU	106.43	2n	53.22	712	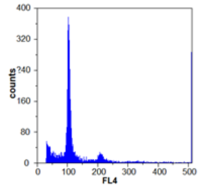
HFTH1	158.77	3n	52.92	708	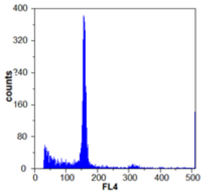

Note: 701Mb genome size of Golden Delicious is from ref. Li et al (2016) as a ref.

I agree that the assembly of HFTH1 represents a notable improvement over Velasco et al (2010) and Li et al (2016), but I think the improvement over the GDDH13 assembly seems incremental.

Response: Thanks for the rigorous suggestion and we totally agree with your opinions. In order to make our description more rigorous, so we have deleted this sentence.

The method to assign centromeres is questionable. The authors may use the algorithm described in https://www.ncbi.nlm.nih.gov/pubmed/23363705 for contromere identification. The conclusion on the evolutionary association of a TE family and centromere based on the presented analysis is most likely speculative.

Response: Thanks for nice suggestions. Actually, we have used previously reported approaches (https://www.ncbi.nlm.nih.gov/pubmed/23363705 and

https://www.nature.com/articles/s41477-018-0166-1) to identity significantly enriched tandem centromeric repeat elements in the HFTH1 genome, but we did not identify any such tandem repeat elements. We have carefully revised this section to avoid some questionable conclusions (lines 153-163).

Unlike human, gorilla and goat, there could be much more variations between genomes of different apple varieties. To confirm gaps of GDDH13 filled by the HFTH1 genome are real gaps in the GDDH13 assembly instead of indels between these two genomes, the authors should align GDDH13 reads to the filled gaps to check the coverage. In addition, could the GDDH13 genome be used to fill gaps in the HFTH1 genome?

Response: Thanks for nice suggestions, we have filtered the closed gaps as your suggestions (see lines 603-607), and 3 gaps closure were removed. We also used the GDDH13 genome to fill gaps for HFTH1 genome and 9 gaps were closed (see lines 179-182).

Genome comparison

Lines 183 and 572-574: First, why Velasco et al was not included to identify shared SNPs? Second, why only shared SNPs were used in the analysis? Many real SNPs that were captured by only one assembly would be missed.

Response: Unable to find and download the short reads of the genome of Velasco et al., we had to use the assembled genome of Velasco et al. to filter specific SNPs in GDDH13 (lines 618-620), because we only want to focus on the difference between 'Golden Delicious' and 'HanFu', and differences between different individuals should be excluded.

Line 184-185, "Approximately 3.45% of non-synonymous SNPs were located within 36.20% of annotated genes": This sentence seems totally wrong. All non-synonymous SNPs should be located in coding regions. Not sure why only 3.45% here? What does "annotated genes" mean? All predicted genes in the genome or only those with

functional annotations?

Response: We have corrected this descriptive error and thank you for your suggestions (lines 193-194).

Line 185-190: Why only top 100 genes were used for enrichment analysis? This does not make any sense. All genes with non-synonymous SNPs should be used. How the authors conclude that "these genes ontologies in 'Hanfu' evolved much faster, providing resistance to various environmental stresses" from this enrichment analysis? I couldn't see any evidence here.

Response: We had re-analyzed this section using all genes with non-synonymous SNPs and rewrote. Besides, because the GO terms for each gene were assigned to the corresponding InterPro entry, but some description of GO terms are obscure for protein/domain functions, such as IPR002182, its corresponding GO term GO:0043531 (ADP binding, https://www.ebi.ac.uk/interpro/entry/IPR002182), while it is a NB-ARC domain. So, we re-enriched using IPR domans (lines 194-197, Supplementary Tables 8 and 9), in order to make the result more straightforward.

Line 192: "(including presence variations) that were with > 100 bp in length" should be "(including presence/absence variations) that were > 100 bp in length".

Response: This has been corrected as suggested (lines 200-201).

Line 194: Based on Fig. 2a, unlike SNPs, the PAV analysis did include the genome of Velasco et al. Why use two different criteria for SNP and PAV analysis? In addition, Fig.2a is very hard to understand. They are not venn diagrams. For example: is "3,287" the number of shared PAVs between Velasco et al and GDDH13 or specific to Velasco et al?

Response: Thanks for nice suggestions. The first question was due to lack of the short reads of the genome of Velasco et al., but we have used the assembled genome of Velasco et al. to filter specific SNPs in GDDH13 (lines 618-620). Besides, we have

modified Fig.2a, so that the results are more explicit (Fig. 2a).

Line 199-202: These two sentences are disconnected. What is the logic to put together "the deletion in 5' UTR of MdCBF2" and "MdCBF2 and other MdCBFs were responsive to low-temperature treatment"?

Response: we removed this sentence as suggested.

Line 220-222: The conclusion here is too speculative. The low diversity in one end of chromosome 5 could be due to introgression and fixation during domestication and breeding process, instead of long-term evolution after WGD.

Response: Thanks for the suggestion and we revised this as suggested.

Dynamic evolution of LTR retrotransposons

The evolution of TEs in apple has been emphasized previously in Daccord et al (2017), and the comparative analyses here in different apple accessions are mostly descriptive and relevant conclusions are likely speculative.

Line 239-242: These statements need more evidence, otherwise it's too speculative.

Response: Thanks for the rigorous suggestion and we totally agree with your opinions. In apple, we try to enrich the dynamic evolution of TEs by the comparative genomic data. But, it is difficult for us to give more evidences in this project. In order to make our description more explicit, so we have deleted this sentence.

Line 246: "ten tested tissues" should be "ten tested samples".

Response: This has been corrected as suggested (lines 255).

Line 253: The proportion of the gene regions plus 5kb flanking regions versus the whole genome should be shown here, otherwise 62.05% may not be a significant value.

Response: Thanks for the suggestion, we have added more details about this section (lines 260-267, Fig. 3e).

Lines 259-260: Fig S14a (box plots) shows the expression as Log (No. of mapped reads). Normalization is needed here. In addition, the statement “indicating that most specific LTR-RTs…gradually weakened” is completely speculative and is not even supported by the data, as in Fig S14a, TE expression is not significantly different in all samples from HFTH1.

Response: Thanks for the suggestion. We attempted to do normalization, but it is difficult to identify the exact exon length of each LTR, because the complete reverse-transcriptase domain sequences cannot be detected in lots of highly diverged LTR-RTs. So, using the count of mapped reads to represent the expression level of each LTR-RT, we think it is ok because we only compared the expression level of each LTR-RT within each sample, and not compare the expression level in different samples, as a similar method is used in https://www.nature.com/articles/ng.3435 (Supplementary Figs. 1, 2 and 3). For another question, we studied and discussed this comment carefully and totally agree, it requires more analysis and data to study the evolutionary of transposons as the divergence time of LTR-RTs increased, so we deleted this statement to avoid misleading readers.

Line 262-270: Indel-associated mutations have been widely reported in plants and animals. This part just confirms findings from other studies without providing much novel information.

Response: Thank you and we have checked our findings in previous published papers. We agree that “Indel-associated mutations have been widely reported in plants and animals”, but the trend of mutation rate as the divergence time increased did not report by any published papers (Fig. 3f, g), we have revised this sections and simplified the description of previous reported results.

Lines 276-277, "deletion events might have made a greater impact than insertions did during the evolution of TEs": deletions and insertions are relative depending on which genome is used as the reference. "deletions" in HFTH1 mean "insertions" in GDDH13. Therefore, this general conclusion is totally wrong.

Response: This statement is incorrect. Because here we defined 'Insertion' as if two copies of TSD flanking the LTR-RT in the HFTH1 genome while only one TSD (no intervening sequences) existed at the corresponding site of the GDDH13 genome. The insertion events are not equal to deletion events in the GDDH13 genome, if a LTR-RT was deleted in the GDDH13 genome, it means this LTR-RT was inserted after the divergence of 'Hanfu' and 'Golden Delicious, this will result in two TSDs (Partial sequence of this LTR-RT has been deleted or replaced with other TE sequences.) or No TSDs (The LTR-RT with two flanking sequences has been deleted.) existed at the corresponding inserted site of the GDDH13 genome. 'Deletion' was defined in two cases: (1) if two TSDs exist at the corresponding inserted site of the GDDH13 genome and the intervening sequence between two TSDs with length less than 100 bp or had no blast hits (BLASTN e-values ≤ 10) to the corresponding LTR-RT sequence in the HFTH1 genome. Partial sequence of this LTR-RT has been deleted or replaced with other TE sequences. (2) No TSDs exist at the corresponding inserted site of the GDDH13 genome. The LTR-RT with two flanking sequences has been deleted. Besides, in order to distinguish from 'deletion and insertion' events defined in previous SV analysis, we have modified the term 'deletion' in this section to 'excision'.

Line 278-279: This statement is too speculative with very weak evidence support.

Response: We have modified this sentence and make it more consistent with our results (most of genomic variation were often associated to TE mobility and TE mobility also caused lot of mutations accumulated in the sequences surrounding TEs. See lines 290-291).

Retrotransposon as an enhancer of *MdMYB1*

The role of MYB1 in regulating anthocyanin biosynthesis has been reported in apple and other plants, and transcriptional regulation of MYB1 by transposon insertion has also been report in other plants (e.g., https://www.ncbi.nlm.nih.gov/pubmed/15143274; https://www.ncbi.nlm.nih.gov/pubmed/20855520); therefore, data presented here, despite being interesting to some degree, mainly confirms similar mechanisms found in other plants. However, my major concern here is that most conclusions are lack of support with strong evidence.

Line 292-293: First, I could not find this conclusion from ref 15. Second, if this is the case, have you check the MdMYB1 allele in HFTH1? A multiple sequence alignment of different alleles should be provided? What if the MdMYB1 allele in HFTH1 contains additional base differences not found in the three reported alleles?

Response: This conclusion is from ref. 13. (Takos, A. M. et al. Light-induced expression of a MYB gene regulates anthocyanin biosynthesis in red apples. Plant Physiol. 142, 1216–1232 (2006).), not from ref. 15. Here, thank you for pointing out the error. In view of the homogous of HFTH1, we sequenced the Hanfu alleles, and carried out two sequence alignment of HFTH1 and Cripps'RedMYB1-1, the results showed that there's nothing except two bases indel in Cripps' Pink MYB1-1. Takoset al. (2006) isolated three types of MdMYB1 alleles: MdMYB1-1 (red allele, DQ886414)), MdMYB1-2 (no red allele, DQ886415) and MdMYB1-3 (no red allele, DQ886416). A comparison of the genomic sequences of HFTH1 (MdMYB1-1) and GDDH13 (MdMYB1-2) showed a different base (G) in the second intron, but this different base was also found in the sequence of Cripps Pink (MdMYB1-3).

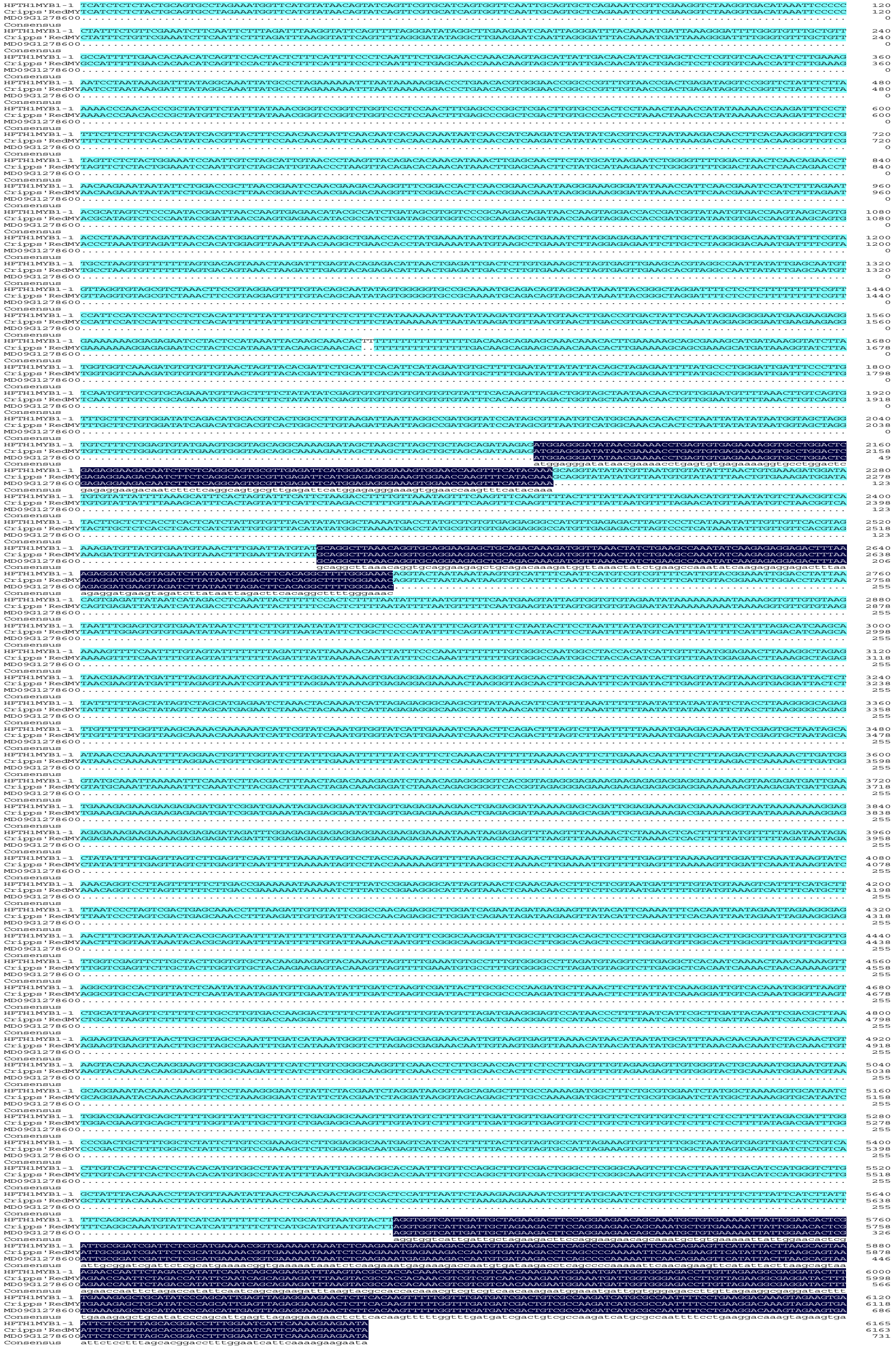

Sequence alignment of HFTH1 and Cripps'RedMYB1-1

Line 297-310: The authors identified several SNPs and two large indels in the

upstream of MdMYB1. How they eliminate the possibility that these SNPs and the other indel could play roles in regulating MdMYB1? I couldn't find the related information from the manuscript. The authors abruptly pick the long insertion for analysis without any reasoning.

Response: First, according to studies from Takos, Adam M., et al. (Takos, Adam M., et al. "Light-induced expression of a MYB gene regulates anthocyanin biosynthesis in red apples." Plant physiology 142.3 (2006): 1216-1232.), we could eliminate these SNPs were not tightly associated with red phenotype. Second, we retrieved resequencing data from the cultivars at GDR (ftp.bioinfo.wsu.edu, Supplementary Table 9) using the junction sequence from the smaller insertions in DDH13. We can get it in red-skinned cultivars 'McIntosh', and PCR amplification was conducted to confirm that the smaller insertions present red and non red-skinned cultivars (see following), these results showed that the smaller insertions may not be related to its phenotype. Additionally, LTR retrotransposons can play crucial roles in tissue-specific expression patterns of pigment genes in plants, which motivated us to pick the long insertion for analysis.

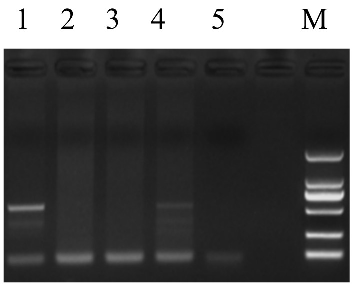

Note: 1, McIntosh; 2, Hhanfu; 3, Huayue; 4, Golden Delicious; 5, Water as template

Line 314-315, "redTE was not found in the non red-skinned accessions": I do find in Table S10 two non-red-skinned accessions ("Roxbury Russet" and "Ningguan") containing redTE.

Response: Thank you for pointing out mistakes for our negligence. So, we had properly modified them, according to our records (Supplementary Figure 11).

Line 322-324: I couldn't follow the logic here. Why talk about flesh color here? It's

OK to conclude that red-flesh in these three accessions is independent of redTE, but the conclusion that it is caused by constitute MdMYB10 expression is highly speculative.

Response: Because red-fleshed apples have a red skin, we wonder whether they possess redTE. ‘constitutive expression of MdMYB10.’ this state quoted from Li, Yuan-Yuan, et al. "MdCOP1 ubiquitin E3 ligases interact with MdMYB1 to regulate light-induced anthocyanin biosynthesis and red fruit coloration in apple." Plant physiology (2012): pp-112. “*MdMYB10* constitutively expressed in the whole plant due to its direct binding to its own enhancer promoter in an autoregulatory-loop manner, leading to the production of red-skinned apple fruit.”, Here, we also revised as: suggesting that the red skin of red-fleshed apple is caused by the constitutive expression of *MdMYB10* binding to R6 motifs of its own promoter in an autoregulatory-loop manner, independent of redTE.

Line 331-335, Figure S15: I am confused here. Why ‘Hanfu’ (red stripe) and ‘HanM’ (fully red) were used here for the analysis? The region MR8-MR11 corresponds to redTE, which was heavily methylated in both ‘Hanfu’ and ‘HanM’. Then how they conclude methylation could be associated with red phenotype? Did the author mean the methylation changes of MR3 and MR7 were induced by redTE? If this is the case, then I did not see any evidence provided.

Response: We understand the confusion surrounding this issue for our ambiguous description, and we revised ‘methylation could be associated with red phenotype?’ as ‘methylation could be associated with variable pattterns of red colour in apple. Hanfu and Hanm were chose for the analysis, because they are the parents of HFTH1, and cytosine methylation is known to be associated with transposons and to influence gene expression (Song, Xianwei, and Xiaofeng Cao. "Transposon-mediated epigenetic regulation contributes to phenotypic diversity and environmental adaptation in rice." Current opinion in plant biology 36 (2017): 111-118.), in order to investigate if DNA methylation in HANFU and mutant HANFUM correlate with

variable pattterns of red colour in apple. Futhermore, a particularly interesting problem remains to investigate how redTE mediated genetic and dynamic epigenetic variation contributes to fruits colouring in response to environmental changes.

Line 336-343: This is purely computational prediction without any experiment evidence supporting the conclusion made here.

Response: This exactly is a result of computational prediction. According to your suggestion, we selected two transcription factor MdHY5, and MdCBF2 for further analysis. We tried to perform an experimental supplement by yeast one-hybrid experiments to test whether MdHY5 is able to bind the predicted elements of redTE, but there was self-activation when target fragments of redTE was fused to pAbAi for bait vector construction. Also, we tried to do it by EMSA, but GST-MdHY5 fusion protein was not detected in our prokaryotic expression system under different induction condition. Here, our EMSA result showed that MdCBF2 could bind the target motif of redTE, see lines 378-386 and Figs. 4d, e.

Line 360-373: Ref 34 describes the identification of a TE using differential display between Jonagold and its color mutants. Authors from this paper never concluded that "red apples have arisen from non red-skinned apples by transposon-induced mutation". Discussion in the section is highly speculative and I couldn't find any of their data to support their speculations. For example, I could not find expression data to support redTE as "a tissue-specific enhancer".

Response: We agree that the citation here is incorrect. In the revised manuscript, we had rewrited. Although enhancer is usually defined as cell or tissue-specific regulatory elements in textbook, we deleted it, in order to make this paper more rigorous.

Lines 382-383: Such strong conclusion can only be made if the full "redTE" sequences are available for all mentioned apple accessions.

Response: We cloned and sequenced the redTE of four red-skinned cultivars ['Cox's Orange Pippin', 'Red Delicious', 'Jonathan' and 'McIntosh'], and alignments

sequence showed they were identical. Still, we removed the “same” as suggested.

Line 384-387: As described in Ref 6, M. sieversii in Xinjiang has not been involved in the domestication process. Therefore, this statement is not correct.

Response: This M.sieversii from Xinjiang wild apple forest do have redTE that was confirmed by sequencing, in accordance with its red phenotype as shown in the picture below or report (WANG Da-jiang et al.,). Here, although the Ref 6 pointed out M. sieversii in Xinjiang has not been involved in the domestication process, if so, we speculate that red trait is likely inherited from its progenitor *M. sieversii* in Xinjiang, and it’s probable that early human activities help to spread *M. sieversii with* eye-catching red apple. In fact, many soft apple domesticated from xinjiang wild apple, such as Xiangguo and Huacaiping et al., with red peel, have been cultivated more than two thousand years in China (Duan, Naibin, et al. "Genome re-sequencing reveals the history of apple and supports a two-stage model for fruit enlargement." Nature communications 8.1 (2017): 249; Da-Jiang, Wang, et al. "Preliminary Investigation of Modern Distribution of Malus Resources in China." Journal of Plant Genetic Resources (2017).), they contain redTE. These results indicated apple from origin center might once have spread both west and east along the old Silk Road, in accordance with breeding and geographical investigations of the origins and history of domesticated apple.

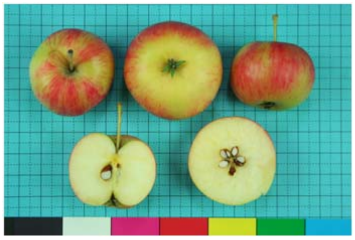

M. sieversii in Xinjiang

Line 406-411: Another speculation. A lot of new data need to be generated to support the TE-regulated expression of MdACS3 and the usefulness of the said marker.

Response: Due to the lack of precise phenotype data, we removed this speculation.

Special thanks to you for your good commends.

Reviewer #3 (Remarks to the Author):

In the manuscript entitled “A high-quality apple genome assembly reveals a retrotransposon controlling red fruit colour” by Zhang et al the authors present a large data set further refining the apple genome, from its first published draft, to a more highly resolved resource. They then focus on one trait - skin color - to illustrate the versatility of having this new detail.The final assembly has very impressive statistics - similar or better than model plant genomes.

The paper is well written and the data is convincing.I have a few issues with the way the paper introduces the work. Firstly it wasn’t until the first section of the results, that I realised the genome wasn’t a double haploid or some other trick of homozygosity. Somehow the authors should state clearly earlier what was done to make HFTH1, and why it lacks much of the heterozygosity that apple usually has. Response: Thanks for nice suggestions, we have now clarified the origin of HFTH1 in Fig. 1a-c. A brief description in lines 70-74 in the introduction section.

The method of anther culture allows a single-step development of homozygous line from heterozygous parent. In general, anther-cultured plants present different kinds of ploidy (2n, 3n, 4n) after undergoing a spontaneous chromosome duplication during in vitro culture, but all of them usually have a haploid origin (M Höfer. "Analysis of simple sequence repeat markers in homozygous lines of apple." Plant Breeding 121.2(2010):159-162; Zhang, Li Yi, et al. "Analysis of Ploidy and Homozygous Genotype of Apple Plants Obtained by Anther Culture." Acta Horticulturae Sinica (2007)). Homozygous tri-haploids HFTH1 (3n) was obtained from the anther culture of heterozygous parents ‘Hanfu’. These results were confirmed by SSR markers and k-mer spectrum analysis as indicators of the homozygosity, but may produce some mutations during in vitro culture, so HFTH1 lacks much of the heterozygosity that apple usually has.

Line 65 - the genetic basis of apple skin color is well understood - perhaps not the mutation that drives yellow verses green skin.

Response: Although, the studies on anthocyanin biosynthesis has been mounting, the mechanism behind the developmental regulation of anthocyanin biosynthesis in fruits remains limited understood. For example, *MdMYB1* is the critical light-inducible TF for anthocyanin biosynthesis. However, how *MdMYB1* is transcriptionally regulated is still not clear. In apple, colour mutation is a complex and intriguing problem. Fruit skin colour was determined by the ground colour and over colour (anthocyanin pigmentation which was superimposed). Ground colour (yellow verses green skin) and over colour of the apple skin were controlled by major gene separately. Therefore, it is perhaps not the mutation caused by redTE that drives yellow verses green skin.

Line 91 - why was HFTH1 more homozygous?

Response: Because HFTH1 is the first generation after a spontaneous chromosome duplication during in vitro anther culture that allows a single-step development of homozygous line from heterozygous parents ("Recovery and characterization of homozygous lines from two sweet orange cultivars via anther culture." Plant Cell, Tissue and Organ Culture (PCTOC) 123.3(2015):633-644.). While GDDH13 was obtained from DH lines after selfing two times according to the GDDH13 genome paper. Besides the exsiting time of HFTH1 (2009) is relatively short, when compared to GDDH13 (1988), which may accumulated some mutations. The result was consistent with our analysis using SSR, k-mer spectrum analysis, heterozygous SNPs detection (see lines 87-94).

Line 228-246 - the difference between LTR-RTs in pear (40) and apple (7800) is very impressive - are the authors certain? Could there have been some masking done in the pear assembly?

Response: Because we only identified intact LTR-RTs, most of intact LTR-RTs cannot

be completely covered in the pear genome because of the fragmented genome assembly (contig N50 of 33.76 kb). On the other hand, most intact LTR-RTs in the apple genome may be inserted after the divergence of apple and pear by comparing the average insert time (0.8 MYA) of these LTR-RTs (lines 242-249) to the divergence time between apple and pear.

Discussion - some mention of the Ruby gene in citrus would be good - there is a transposon that drives expression of the citrus MYB during the cold in orange - could expression out of the apple LTR-RT be cold induced?

Response: In apple, light is an essential prerequisite of fruit colouring (Steyn, Willem J., et al. "Evidence for a photoprotective function of low‐temperature‐induced anthocyanin accumulation in apple and pear peel." Physiologia Plantarum 136.4 (2009): 461-472; Ubi, Benjamin Ewa, et al. "Expression analysis of anthocyanin biosynthetic genes in apple skin: effect of UV-B and temperature." Plant Science 170.3 (2006): 571-578.). Without light, then even low ambient temperatures could not induce the expression of *MdMYB1*, which differs from the cold-induced *Ruby* gene in citrus. However, in the sunshine condition, a relatively low temperature can enhance the colouration of fruits ,which is presumably regulated via recruitment of other transcription factors, such as MdCBF2 et al. Based on RNA-seq, a low-temperature-inducible transcription factor MdCBF2 (MD06G1072200) was chosen for its binding analysis by electrophoretic mobility shift assay（EMSA）.Our result confirmed that MdCBF2 was capable of binding to the sequence (GCCGACTT) (Supplementary Figure16), indicating it was involved in fruit coloration via redTE regulatory networks under relative low ambient temperature. Here, special thanks to you for your good commends.

REVIEWERS' COMMENTS:

Reviewer #1 (Remarks to the Author):

The authors have addressed most of my comments. Here some points to consider:
Line 179: Indicate the number of bases used to fill the gaps of HFTH1
Lines 283-289: Retrotransposons do not "excise" (in contrast to DNA transposons), they get eliminated by homologous recombination. That is why some sequence is still left in GDDH13 for instance.

Globally the role of redTE in the control of fruit colour still needs to be toned down. Especially since the authors do not observe the expected co-expression of the TE with the downstream gene. So far the authors only present correlations. therefore e.g. line 377 should state: "Given that redTE may control MdMYB1..". and line 386 "indicating it is potentially involved in fruit coloration via redTE regulatory networks"

413-418: numerous typos

supplementary tables 11 and 12 are missing!

437: This is not surprising

Generally, the newly added text requires intensive controls for spelling and english errors.

Reviewer #2 (Remarks to the Author):

The authors have addressed most of my concerns and this revised manuscript is much improved. I appreciate the effort and especially the newly added data and analysis to support the role of redTE in fruit skin coloration. However, I still have two major concerns:

1) The manuscript is not well written. There are quite a lot of syntax and grammar errors. The entire manuscript should be revised by native English speakers.

2) Line 311-312, “Besides some SNPs and two larger insertions in the upstream regulatory region of this gene were found”: The authors provided a gel picture in the response letter to suggest that the 501-bp insertion in the GDDH13 genome is not associated with fruit skin color. This result should be included in the manuscript. The authors mentioned that SNPs in this region are not associated with red phenotype according to Takos et al. (http://www.plantphysiol.org/content/142/3/1216). I am sorry that I could not find the related text from this paper. The authors may use the genome resequencing data reported in Duan et al. (https://www.nature.com/articles/s41467-017-00336-7) to see the patterns of these SNPs in different accessions and rule out the possibility of their associations with fruit skin color. In addition, how many SNPs were identified in this region? “some” should be replaced by a number.

Minor:
3) Line 101-108: “Gap filling” should be mentioned here. Otherwise, it’s hard to understand how to get two very different contig N50 sizes (4.63 Mb and 6.99 Mb) as polishing and scaffolding using BioNano maps and Hi-C data should not change the contig N50 size, at least not too much.

Reviewer #3 (Remarks to the Author):

The authors have adequately addressed most of my suggested changes and edits.
However - for context - it would still be good to mention citrus anthocyanins being induced by cold (because of a transposon). This is not my work (!) but the common observation does provide some exciting possibilities for other crops where colour is elevated in the cold.
Also - the statement that "how MdMYB1 is transcriptionally regulated is still not clear. " is a little inaccurate - there are a number of upstream regulators reported - MYB1 itself, HY5, MYB23 activates CBF2, COL11.

Authors' point-by-point response to the reviewers

Title: A high-quality apple genome assembly reveals the association of a retrotransposon and red fruit colour

Response to the Reviewers:

We are genuinely appreciative of the Editor for inviting us to submit a revised manuscript. We thank the three reviewers for thoughtful recommendations of this work. We have made the polished changes requested by the reviewers. All changes are highlighted in the manuscript text (in WORD.DOC) file.

Reviewer #1 (Remarks to the Author):

The authors have addressed most of my comments. Here some points to consider:

1) Line 179: Indicate the number of bases used to fill the gaps of HFTH1

Response: we have added more details about this. We filled 9 genomic gaps with an average length of 42,368 bp for the HFTH1 genome using the GDDH13 genome.

2) Lines 283-289: Retrotransposons do not "excise" (in contrast to DNA transposons), they get eliminated by homologous recombination. That is why some sequence is still left in GDDH13 for instance.

Response: This has been corrected as suggested.

3) Globally the role of redTE in the control of fruit colour still needs to be toned down. Especially since the authors do not observe the expected co-expression of the TE with the downstream gene. So far the authors only present correlations. therefore e.g. line 377 should state: "Given that redTE may control MdMYB1..". and line 386 "indicating it is potentially involved in fruit coloration via redTE regulatory networks".

Response: we quite agree with your views. Indeed, in our current studies, the evidences are mostly limited to correlative observations on the basis of association of redTE with nearby gene with biologically functions plausible link to phenotype.

Therefore, in order to get more scientific and rigorous conclusion, we have used a modified title as "A high-quality apple genome assembly reveals the association of a retrotransposon and red fruit colour'' in the new manuscript. A recent studies have showed gene editing methods can directly test the TE function. The next step, we will carry out CRSPR-mediated deletion to determine redTE function. Besides, the states in line 377 and line 386 have been revised as suggested.

4) 413-418: numerous typos

Response: Numerous typos in manuscript have been revised.

5) Supplementary tables 11 and 12 are missing!

Response: supplementary tables 11 and 12 were provided with supplementary file.

6) 437: This is not surprising

Response: We have replaced 'Surprisely' with 'Interestingly'.

7) Generally, the newly added text requires intensive controls for spelling and English errors.

Response: The manuscript had been revised by native English speakers. Here, special thanks to you for your nice suggestions again.

Reviewer #2 (Remarks to the Author):

The authors have addressed most of my concerns and this revised manuscript is much improved. I appreciate the effort and especially the newly added data and analysis to support the role of redTE in fruit skin coloration. However, I still have two major concerns:

1) The manuscript is not well written. There are quite a lot of syntax and grammar errors. The entire manuscript should be revised by native English speakers.

Response: Thank the reviewer for the constructive suggestions. The manuscript had been revised by native English speakers.

2) Line 311-312, “Besides some SNPs and two larger insertions in the upstream regulatory region of this gene were found”: The authors provided a gel picture in the response letter to suggest that the 501-bp insertion in the GDDH13 genome is not associated with fruit skin color. This result should be included in the manuscript. The authors mentioned that SNPs in this region are not associated with red phenotype according to Takos et al. (http://www.plantphysiol.org/content/142/3/1216). I am sorry that I could not find the related text from this paper. The authors may use the genome resequencing data reported in Duan et al. (https://www.nature.com/articles/s41467-017-00336-7) to see the patterns of these SNPs in different accessions and rule out the possibility of their associations with fruit skin color. In addition, how many SNPs were identified in this region? “some” should be replaced by a number.

Response: we performed a sequence alignment of MdMYB1 in the HFTH1 and GDDH13 genomes. The results showed that the coding sequences of MdMYB1 were identical, but one SNP was detected in the intron regions. In addition, fifteen SNPs and five indels in the upstream region were found.

In our studies, we were able to rule out 16 SNPs (including a SNP in intron region) and 3 indels that are not correlated with fruit skin colour using the sequencing data(AB557640, AB557638, DQ886416, DQ886414, DQ886415, EU518249, HQ259417, KX822763) from Genbank and reported by Takos et al.(http://www.plantphysiol.org/content/142/3/1216) , and re-sequencing data at GDR (https://www.rosaceae.org/) and reported in Duan et al. (https://www.nature.com/articles/s41467-017-00336-7). But one 2-bp indel located in 17 repeat T bases in the HFTH1 genome have a complex polymorphism in red apple accessions. See as Supplementary Table 10.

3) Line 101-108: “Gap filling” should be mentioned here. Otherwise, it’s hard to understand how to get two very different contig N50 sizes (4.63 Mb and 6.99 Mb) as polishing and scaffolding using BioNano maps and Hi-C data should not change the contig N50 size, at least not too much.

Response: We added a sentence in the main text to properly state “58.5% gaps introduced in this step were closed by subsequent gap filling procedure.”, Thank you for your valuable suggestions!

Reviewer #3 (Remarks to the Author):

1) The authors have adequately addressed most of my suggested changes and edits. However - for context - it would still be good to mention citrus anthocyanins being induced by cold (because of a citrus). This is not my work (!) but the common observation does provide some exciting possibilities for other crops where colour is elevated in the cold.

Response: In context, we have cited this classic example of citrus identified a copia-like retrotransposon as functional cold-inducible element, which exactly provides some exciting possibilities for other crops where colour is elevated in the cold as your suggestions.

2) Also, the statement that "how MdMYB1 is transcriptionally regulated is still not clear." is a little inaccurate - there are a number of upstream regulators reported - MYB1 itself, HY5, MYB23 activates CBF2, COL11.

Response: Now, we have realized that some of the descriptions in the previous response-letter ‘how MdMYB1 is transcriptionally regulated ‘was really not so accurate and a little bit arbitrary. Thank you for offering such a valuable cues about the transcriptionally regulated network of *MdMYB1*, and we will keep on making efforts to do it better in future.
